# CCDC88B interacts with RASAL3 and ARHGEF2 and regulates dendritic cell function in neuroinflammation and colitis

**DOI:** 10.1038/s42003-023-05751-9

**Published:** 2024-01-10

**Authors:** Jean-Frederic Olivier, David Langlais, Thiviya Jeyakumar, Maria J. Polyak, Luc Galarneau, Romain Cayrol, Hua Jiang, Kelly R. Molloy, Guoyue Xu, Harumi Suzuki, John LaCava, Philippe Gros, Nassima Fodil

**Affiliations:** 1 Department of Biochemistry, McGill University, Montreal, QC Canada; 2 McGill Research Center on Complex Traits, Montreal, QC Canada; 3 Department of Human Genetics, Victor Phillip Dahdaleh Institute of Genomic Medicine, Montreal, QC Canada; 4 Department of Medicine, Sherbrooke University, Sherbrooke, QC Canada; 5 Department of Pathology, University of Montreal Hospital Center (CHUM), Montreal, QC Canada; 6 University of Montreal Hospital Center Research Center (CR-CHUM), Montreal, QC Canada; 7 Department of Pathology and Cellular Biology, University of Montreal, Montreal, QC Canada; 8 Laboratory of Cellular and Structural Biology, The Rockefeller University, New York, NY USA; 9 Laboratory of Mass Spectrometry and Gaseous Ion Chemistry, The Rockefeller University, New York, NY USA; 10 Department of Immunology and Pathology, National Center for Global Health and Medicine, Tokyo, Japan; 11 European Research Institute for the Biology of Ageing, University Medical Center Groningen, Groningen, The Netherlands; 12 CERMO-FC, Pavillon des Sciences Biologiques, Montreal, QC Canada

**Keywords:** Cell migration, Inflammation, Protein-protein interaction networks, Inflammatory bowel disease

## Abstract

*CCDC88B* is a risk factor for several chronic inflammatory diseases in humans and its inactivation causes a migratory defect in DCs in mice. CCDC88B belongs to a family of cytoskeleton-associated scaffold proteins that feature protein:protein interaction domains. Here, we identified the Rho/Rac Guanine Nucleotide Exchange Factor 2 (ARHGEF2) and the RAS Protein Activator Like 3 (RASAL3) as CCDC88B physical and functional interactors. Mice defective in *Arhgef2 or Rasal3* show dampened neuroinflammation, and display altered cellular response and susceptibility to colitis; *ARHGEF2* maps to a human Chromosome 1 locus associated with susceptibility to IBD. *Arhgef2* and *Rasal3* mutant DCs show altered migration and motility in vitro, causing either reduced (*Arhgef2*) or enhanced (*Rasal3*) migratory properties. The CCDC88B/RASAL3/ARHGEF2 complex appears to regulate DCs migration by modulating activation of RHOA, with ARHGEF2 and RASAL3 acting in opposite regulatory fashions, providing a molecular mechanism for the involvement of these proteins in DCs immune functions.

## Abstract (web summary)

CCDC88B physically interacts with ARHGEF2 and RASAL3; defective mice show dampened neuroinflammation, altered susceptibility to colitis and altered DCs motility by modulating RHOA, with ARHGEF2 and RASAL3 acting in opposite regulatory fashions.

## Introduction

Neuroinflammation refers to inflammation of the brain and of the spinal cord. Acute neuroinflammation is a severe and rapidly fatal condition encountered in microbial or auto-immune encephalitis^1,2^. Neuroinflammation is a common feature and contributor of chronic inflammatory, autoimmune and neurodegenerative diseases affecting the brain and the spinal cord such as in multiple sclerosis (MS), Alzheimer’s disease and Parkinson’s disease^3^. Chronic neuroinflammation is a major driver of pathogenesis in these conditions, and a better understanding of the underlying cellular and molecular mechanism is required to develop new treatment strategies. The mouse model of experimental cerebral malaria (ECM) induced by infection with *Plasmodium berghei* ANKA (PbA) has been used extensively to demonstrate the critical role of myeloid cells (neutrophils, dendritic cells, macrophages), CD4^+^ and CD8^+^ T cells and NK cells in the pathogenesis of acute neuroinflammation, and to establish the role of pro-inflammatory mediators (e.g., TNF, type I and type II IFN, IL1b, MIP1a, MIP1b/CCL4, CXCL1, CXCL9, CXCL10)^4^. On the other hand, the mouse model of experimental autoimmune encephalomyelitis (EAE) has been used to study cell types and molecules involved in chronic neuroinflammation with associated autoimmunity^5^.

We have used genome wide mutagenesis in mice to identify novel genes that modulate neuroinflammation, and for which mutational inactivation protects against lethal encephalitis during infection with *Plasmodium berghei* ANKA^6–9^. One of the genes uncovered was *Ccdc88b*, a gene expressed exclusively in hematopoietic organs (spleen, bone marrow, lymph nodes and thymus), and in lymphoid and myeloid cells derived from them^6^. Immunophenotyping studies indicated that abrogation of *Ccdc88b* function causes loss of T lymphocyte function, with decreased maturation, impaired activation and reduced cytokine production in response to pro-inflammatory signals or T-cell receptor engagement^6^. In myeloid cells, loss of *Ccdc88b* impairs several aspects of dendritic cell (DC) function, suggesting that the CCDC88B protein is essential for cellular and molecular pathways common to these two cell types. Finally, we have shown that CCDC88B is required for in vitro mobility and in vivo migration of DCs, including their capacity to activate T lymphocytes^10^.

In humans, the *CCDC88B* gene maps on Chromosome 11q13 within a locus associated with vulnerability to several common inflammatory diseases including psoriasis, primary biliary cirrhosis, sarcoidosis, MS, and inflammatory bowel disease (IBD)^11–13^. In mouse models, we have shown that *Ccdc88b*-deficient mice are protected against DSS-induced intestinal colitis. Likewise, in a T cell transfer model, *Ccdc88b*-deficent T cells do not induce colitis in immunocompromised *Rag1*^*−/−*^ mice^13^. Parallel studies have shown that the CCDC88B protein and mRNA are elevated in the colon of IBD patients. Furthermore, expression of *CCDC88B* mRNA is regulated by cis-acting variants in CD14^+^ myeloid cells, with *CCDC88B* mRNA expression correlated positively with disease risk in a cohort of Crohn’s disease patients^13^. Together, these findings have established a key role for CCDC88B in the pathogenesis of inflammatory conditions of the brain and of the gut.

CCDC88B belongs to the Hook-related protein family that includes, CCDC88A (GIV, Girdin, HkRP1), CCDC88B (Gipie, HkRP3) and CCDC88C (DAPLE, HkRP2). These Hook proteins are defined by shared secondary structure motifs that include an N-terminal Hook-related microtubule-binding domain (MBD), a central coiled coil domain (CCD), and a C-terminal domain involved in binding different subcellular organelles^14^. Whereas the MBD and CCD domains show sequence conservation, the divergent C-terminal domain is thought to confer protein-specific functions^14,15^. The study of these proteins has shown that they are cytoplasmic scaffold proteins that function in different physiological and biochemical pathways, some of which have been revealed by proteins they recruit to specific molecular scaffolds. CCDC88A binds to the Akt kinase and to the actin cytoskeleton and is involved in migration of cancer cells, and in angiogenesis^16^. CCDC88C is a cytoplasmic scaffold protein essential for transducing Wnt signaling pathways^17^, and mutations in *CCDC88C* have been linked to transformation and metabolism of cancer cells^18,19^; familial autosomal recessive truncations of CCDC88C was also found in severe congenital hydrocephalus^20^. CCDC88B was shown to interact with the CDC42 guanine nucleotide exchange factor DOCK 8^21^.

Here, we have used a systematic proteomics approach to identify CCDC88B protein interactors in primary thymocytes and in the T cell line BI-141. These studies identified the Rho/Rac Guanine Nucleotide Exchange Factor 2 (ARHGEF2) and the RAS Protein Activator Like 3 (RASAL3) as specific CCDC88B interactors, and this was further validated by co-immunoprecipitation and by double immunofluorescence with confocal microscopy. In addition, mice defective in *Rasal3* or *Arhgef2* share a protective phenotype in mouse models of inflammation similar to that exhibited by *Ccdc88b* mutants: *Arhgef2* and *Rasal3* deficient mice are protected against neuroinflammation in the EAE model and, to a lesser extent, the ECM model. Interestingly, loss of *Rasal3* or *Arhgef2* also exacerbates the effect of DSS induced colitis. Finally, mutations in *Rasal3* and in *Arhgef2* affect the motility and migration of DCs, a function also affected in *Ccdc88b* mutant primary DCs^10^. These studies identify the CCDC88B/RASAL3/ARHGEF2 molecular scaffold as playing a critical role in the migratory properties of DCs.

## Results

### RASAL3 and ARHGEF2 physically interact with CCDC88B

We previously established the cellular basis for the ECM and IBD protective effect of *Ccdc88b* inactivation^13^. Yet, the underlying mechanistic basis of these effects remains unknown. Primary sequence analysis of CCDC88B points to several family-specific sequence motifs associated with protein-protein interaction and organelles binding or localization. To identify CCDC88B-dependent biochemical pathways important for the function of myeloid and lymphoid cells, we sought to identify proteins that physically and functionally interact with CCDC88B in these cells^6,10,13^.

**Fig. 1:**
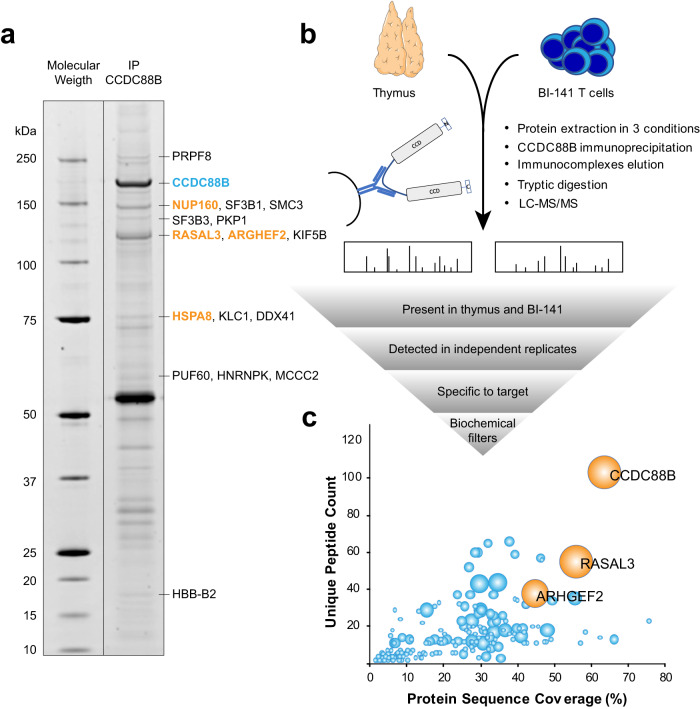
Identification of CCDC88B-interacting proteins by LC-MS/MS. **a** Co-immunoprecipitation of CCDC88B from whole thymus extract with anti-CCDC88B anti-serum analyzed by gel electrophoresis. The gel was stained with Sypro, nine bands were excised and submitted to LC-MS/MS (see “Results”). The four most abundant proteins (spectral count >20) are identified in orange. **b** Schematic representation of the experimental procedure used for proteomics analysis. Proteins were extracted from whole thymus or BI-141 T cell line using three different lysis buffers, then submitted to immunoprecipitation using an anti-CCDC88b polyclonal anti-serum. Eluates were subjected to LC-MS/MS to identify and quantify enriched proteins. The results were analyzed using multiple filtering parameters to identify the most probable CCDC88B interactors. An independent experiment was performed in duplicate on whole thymic extracts. **c** Graph presenting LC-MS/MS quantitative results. The percentage of protein length that was covered by MS/MS identified peptides is plotted against the number of unique peptides identified, whereas the bubble size is proportional to a score (arbitrary units) based on filtering parameters (see Supplementary Data 1); the two best candidates (along with CCDC88B) are drawn in orange. Parts of (**b**) were drawn by using pictures from Servier Medical Art. Servier Medical Art by Servier is licensed under a Creative Commons Attribution 3.0 Unported License (https://creativecommons.org/licenses/by/3.0/).

We first performed an experiment where CCDC88B was immunoprecipitated from murine thymic extracts, followed by separation of captured proteins by SDS-PAGE. Nine Sypro stained bands were excised and analyzed by LC-MS/MS (Fig. 1a and Supplementary Data 1), and four different putative CCDC88B interactors were detected with a high spectral count: RAS Protein Activator Like 3 (RASAL3), Rho/Rac Guanine Nucleotide Exchange Factor 2 (ARHGEF2), Heat Shock Protein Family A Member 8 (HSPA8) and Nucleoporin 160 (NUP160). Of note, these proteins were excised at their corresponding molecular mass and were easily visible on SDS-PAGE. CCDC88B was also detected as the most abundant protein at its expected molecular mass (Fig. 1a).

We next carried out co-immunoprecipitations of CCDC88B from cryo-milled BI-141 T cells and mouse thymi, followed by identification of pulled down proteins by LC-MS/MS (Fig. 1b); 253 proteins were detected (Supplementary Data 1) and filters taking into account abundance (number of peptides and protein coverage), relative intensity, retention of interaction under different co-immunoprecipitation conditions, and presence in both BI-141 cells and in primary thymic co-immunoprecipitates in independent tests were applied. Because CCDC88B is a cytoplasmic protein, nuclear proteins, DNA-binding proteins and species commonly found in protein:protein interaction studies^22^ were discarded. A priority score was assigned to the different species detected (Fig. 1b; Supplementary Data 1 and Supplementary Fig. 1), resulting in the identification of RASAL3 and ARHGEF2 as the two top candidate CCDC88B interactors (Fig. 1c). RASAL3 is a 114.7 kDa cytosolic protein, member of the Ras GTPase-activating proteins (RasGAP) family with characteristic pleckstrin homology (PH), C2, and Ras GTPase-activation protein (RasGAP) domains. This protein family may act as a negative regulator of Ras signaling^23^. ARHGEF2 (also known as GEF-H1) is a 133.7 kDa cytosolic protein member of the Rho/Rac Guanine Nucleotide Exchange Factor family known to activate Rho-GTPases by promoting the exchange of GDP for GTP^24^.

**Fig. 2:**
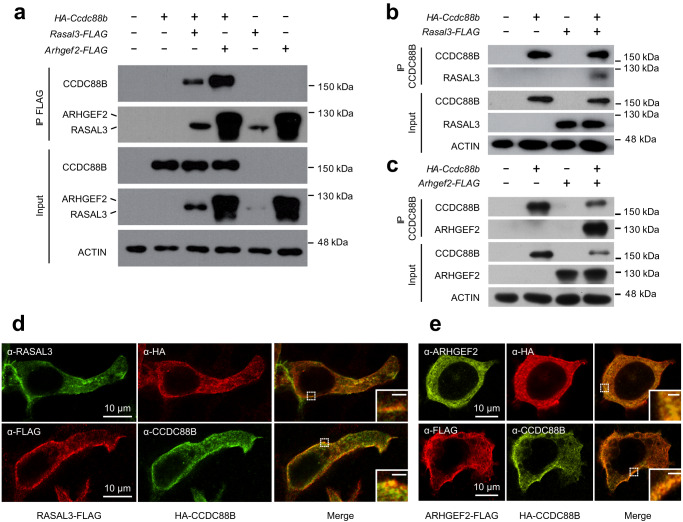
Validation of physical interaction between CCDC88B, ARHGEF2 and RASAL3. **a** Co-immunoprecipitation demonstrating interactions between CCDC88B, RASAL3 and ARHGEF2. HEK293T cells were transiently transfected with the indicated expression vectors and cells extracts were subjected to immunoprecipitation with an anti-FLAG monoclonal antibody. Immunoprecipitated proteins (IP FLAG) and total lysates (Input) were analyzed by gel electrophoresis (SDS-PAGE) and subjected to immunoblotting using an anti-HA monoclonal antibody to reveal HA-tagged CCDC88B. **b**, **c** HEK293T cells transfected with the indicated expression vectors were subjected to immunoprecipitation with an anti-CCDC88B polyclonal anti-serum. Immunoprecipitated proteins (IP CCDC88B) and total lysates (Input) were analyzed by gel electrophoresis and subjected to immunoblotting using an anti-FLAG monoclonal antibody to reveal FLAG tagged RASAL3 and ARHGEF2 (**b**, **c** respectively). **d**, **e** Double immunofluorescence of HA-CCDC88B and RASAL3-FLAG or ARHGEF2-FLAG co-expressed in transiently transfected HEK293T cells, respectively. Tagged proteins were revealed by combinations of anti-FLAG, anti-CCDC88B, anti-HA, and anti-ARHGEF2 (with corresponding fluorophores) as indicated in the boxes. Images were acquired by confocal microscopy, insert scale bar = 1 μm. Closeups in inserts show the extent of colocalization near the cell membrane.

Physical interaction between CCDC88B, RASAL3 and ARHGEF2 was further investigated following pairwise transient expression of the 3 proteins in HEK293T cells (Fig. 2). Cell extracts were subjected to immunoprecipitation and immunoblotting with pairs of antibodies; CCDC88B (detected by immunoblotting with anti-HA) could be pulled down by either RASAL3 or ARHGEF2 when immunoprecipitated with anti-FLAG antibody (Fig. 2a). Conversely, CCDC88B immunoprecipitated both RASAL3 (Fig. 2b) and ARHGEF2 (Fig. 2c) detected by anti-FLAG. In addition, interaction between CCDC88B, RASAL3, and ARHGEF2 was analyzed by double immunofluorescence following expression of the proteins in HEK293T cells transfected as above. These experiments identified a strong overlapping staining between CCDC88B and RASAL3 (Fig. 2d) or ARGHEF2 (Fig. 2e). Staining for the 3 proteins expressed in HEK293T cells was very similar, appearing primarily associated with both the cell membrane and with intracellular punctate/filamentous compartment.

Taken together, these studies strongly suggest that CCDC88B forms a cellular complex with RASAL3 and ARHGEF2 in T cells. This complex appears associated with the cell membrane and with an intracellular reticulated compartment. These findings are in agreement with our recent observation that CCDC88B co-localizes with intracellular actin filaments^10^ and suggest that the CCDC88B/ARHGEF2/RASAL3 complex may be associated with the cytoskeleton.

### Absence of RASAL3 and ARHGEF2 protects against neuroinflammation

**Fig. 3:**
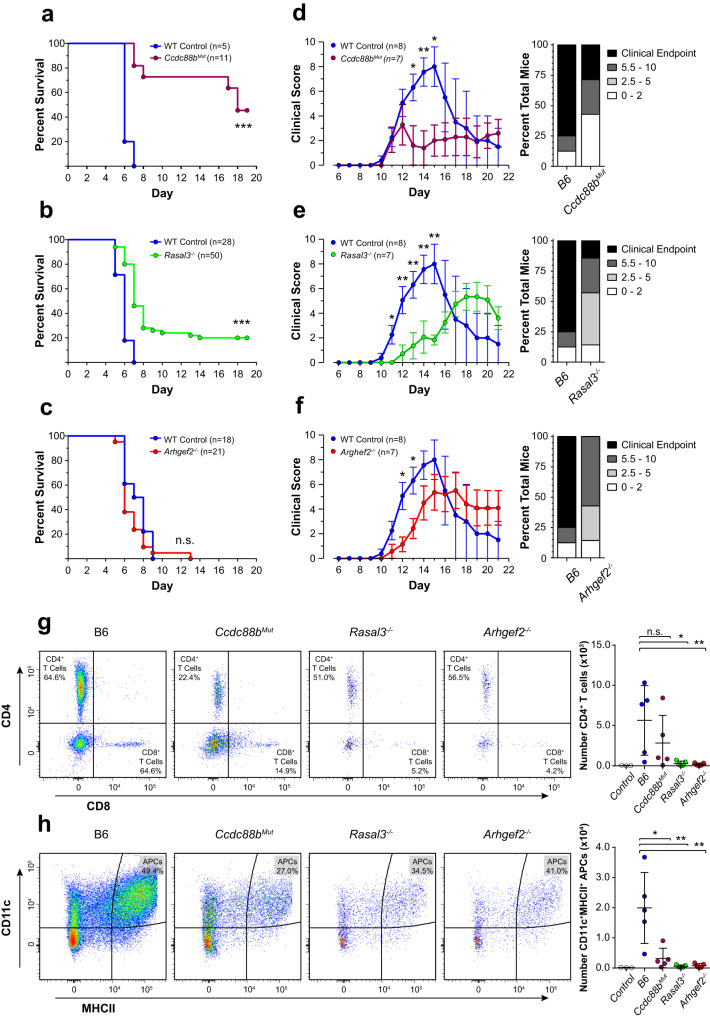
Loss of RASAL3 and ARHGEF2 reduces susceptibility to neuroinflammation. Survival of B6, *Ccdc88b*^*Mut*^ (**a**), *Rasal3*^*−/−*^ (**b**), and *Arhgef2*^*−/−*^ (**c**) mutant mice following infection with *Plasmodium berghei* ANKA (PbA). The total number of mice in each group is indicated; animals surviving beyond day 8 are considered resistant to PbA-induced lethal neuroinflammation (n.s. non-significant, ***p < 0.001, Gehan-Breslow-Wilcoxon test). **d**–**f** Experimental autoimmune encephalomyelitis (EAE) was induced by combined treatment with pertussis toxin and myelin oligodendrocyte protein and the extent of pathogenesis (clinical score) was assessed daily as described in “Materials and methods”. Clinical scores (left) and EAE incidence, representing the highest score reached by each individual mouse over the course of the experiment (right), of B6, *Ccdc88b*^*Mut*^, *Rasal3*^*−/−*^ and *Arhgef2*^*−/−*^ mice are shown, along with the number of mice used in each group (clinical scores shown as means ± SEM; *p < 0.05, **p < 0.01; Mann–Whitney test). Data from (**d**) to (**f**) are representative of at least three independent experiments where each group were compared to the same controls within the same experiment. **g**, **h** EAE was induced in B6, *Ccdc88b*^*Mut*^, *Rasal3*^*−/−*^ and *Arhgef2*^*−/−*^ mice and the spinal cords harvested at day 11. Total CD45^+^CD3^+^CD4^+^ T cells (**g**) and total CD45^+^CD11c^High^MHCII^High^ antigen presenting cells (**h**) were analyzed by flow cytometry. Representative flow cytometry data (normalized for 10 million viable cells; left) and quantification of individual mice (right) for each genotype are shown; data from (**g**) are gated first on total viable CD45^+^CD3^+^ cells and data from (**h**) are gated first on total viable CD45^+^ cells (see Supplementary Fig. 3). Control: B6 mice without EAE induction.

We next tested whether the proximity and physical interaction detected between CCDC88B, ARHGEF2 and RASAL3 translates into a functional relationship. Having previously shown that *Ccdc88b* inactivation protects against neuroinflammation (ECM model)^6^, we evaluated a possible role for ARHGEF2 and RASAL3 in neuroinflammation using mouse mutants inactivated at each locus. For this, we created an *Arhgef2* loss-of-function mutant by CRISPR-Cas9 gene editing in a C57BL/6J (B6) genetic background (Supplementary Fig. 2). *Rasal3*^*−/−*^ mutant mice have been previously described^25^. Control B6, along with *Ccdc88b*^*Mut*^, *Rasal3*^*−/−*^ and *Arhgef2*^*−/−*^ mutant mice were infected with PbA, monitored for appearance of neurological symptoms and survival was recorded (Fig. 3a–c). *Ccdc88b*^*Mut*^ mice were highly resistant to the cerebral phase of the disease (d5–d9), with greater than ~70% survival at d16 (Fig. 3a). On the other hand, *Rasal3*^*−/−*^ mice showed a moderate but significant level of resistance over controls, with ~20% survival beyond d16 (Fig. 3b). *Arhgef2*^*−/−*^ mutant mice were as susceptible to ECM as controls (Fig. 3c).

We also investigated *Ccdc88b*^*Mut*^, *Arhgef2*^*−/−*^ and *Rasal3*^*−/−*^ mutants in a non-microbial model of neuroinflammation (Fig. 3d–f). In EAE, neuroinflammation and axonal damage are induced by autoimmune response to myelin oligodendrocyte glycoprotein which is co-administered with pertussis toxin. Response to EAE is evaluated by appearance and severity of symptoms assessed by a clinical score and by overall survival. In the EAE model, *Ccdc88b*^*Mut*^ (Fig. 3d), *Rasal3*^*−/−*^ (Fig. 3e), and *Arhgef2*^*−/−*^ (Fig. 3f) mutants showed significant protection when compared to B6 controls, including lower clinical scores and increased survival, as identified by the fraction of animals reaching clinical endpoint (Fig. 3d–f). Clinical scores for individual mice revealed that all mutants displayed a much milder phenotype than controls.

To better characterize the basis of EAE resistance seen in *Ccdc88b*^*Mut*^, *Rasal3*^*−/−*^ and *Arhgef2*^*−/−*^ mice, we investigated the infiltration of pro-inflammatory cells in the spinal cord of mutant and controls at day 11, a time point that corresponds to appearance of neurological symptoms (Fig. 3d–f). Flow cytometry analysis (see Supplementary Fig. 3) of the cellular content of *Rasal3*^*−/−*^ and *Arhgef2*^*−/−*^ spinal cord identified a significant reduction in the numbers of infiltrating CD45^+^CD3^+^CD4^+^ T cells (Fig. 3g) and CD45^+^CD11c^+^MHCII^+^ antigen presenting cells (Fig. 3g) compared to B6 controls. The decreased infiltration of antigen presenting cells was also noted in the spinal cord of *Ccdc88b*^*Mut*^ mice (Fig. 3h). These results strongly suggest that EAE resistance in *Rasal3*^*−/−*^, *Arhgef2*^*−/−*^ and *Ccdc88b*^*Mut*^ is associated with reduced/delayed infiltration of lymphoid and myeloid cells in the spinal cord, with possible concomitant dampening of pathological neuroinflammation.

These results show that the *Ccdc88b*^*Mut*^, *Arhgef2*^*−/−*^ and *Rasal3*^*−/−*^ mutants display significant resistance in one or both models of neuroinflammation models tested. Hence, the physical interaction detected between the CCDC88B, ARHGEF2 and RASAL3 proteins appears to underlie functional relationship when tested in vivo during neuroinflammation.

### Rasal3^−/−^ and Arhgef2^−/−^ mice are highly susceptible to DSS-induced colitis

**Fig. 4:**
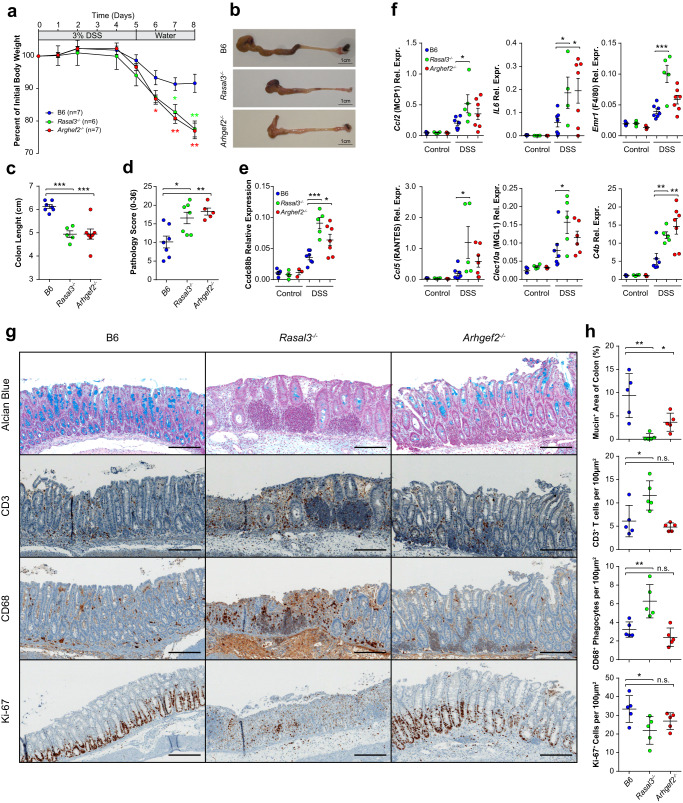
Loss of RASAL3 or ARHGEF2 enhances pathogenesis of DSS-induced colitis. Control B6, as well as *Rasal3*^*−/−*^ and *Arhgef2*^*−/−*^ mutant mice were given 3% DSS for 5 days followed by 3 days of water and were then sacrificed. **a** Body weight loss, expressed as percent of initial weight, for each genotype (means ± SEM, *p < 0.05, **p < 0.01; two-tailed Student’s *t* test). **b** Representative images of colons from B6, *Rasal3*^*−/−*^ and *Arhgef2*^*−/−*^ mice at day 8. **b** Quantification of colons length (means ± SEM. ***p < 0.001; two-tailed Student’s *t* test). Data from (**a**) to (**c**) are representative of three independent experiments. **d** Pathology scores evaluating inflammatory cell infiltration, submucosal edema, gland loss and surface epithelial erosion/ulceration (mean ± SEM; *p < 0.05, **p < 0.01; two-tailed Student’s *t* test). qPCR data for the relative expression for *Ccdc88b* (**e**) as well as indicated genes coding for cytokines, chemokines, and myeloid cell markers (**f**) in distal colons of non-treated (Control) or at day 8 following DSS treatment (DSS). Data represent expression relative to *Hprt* which was used as an internal control (means ± SEM, *p < 0.05, **p < 0.01; ***p < 0.001; two-tailed Student’s *t* test). **g** Immunohistochemistry staining of colons of B6, *Rasal3*^*−/−*^ and *Arhgef2*^*−/−*^ mice at day 8 following DSS treatment for the indicated markers. Insert bar: 100 µm. Data are representative of at least 5 colons per group. **h** Quantification of (**g**), for the average of the two most distal field of view of each colon (at 10X, using automated counting, see “Materials and methods”). Each data point represents an individual mouse (means ± SEM, n.s. non-significant; *p < 0.05, **p < 0.01; two-tailed Student’s *t* test).

We have previously shown that the protection of *Ccdc88b*^*Mut*^ mice against intestinal colitis is due to reduced intestinal inflammation and decreased leukocyte infiltration^13^. We therefore tested the response of *Arhgef2*^*−/−*^ and *Rasal3*^*−/−*^ mutant to DSS-induced colitis. We noticed that both mutant mice lost significantly more weight than the B6, an indicator of the pathology progression (Fig. 4a). Moreover, the colons from both *Arhgef2*^*−/−*^ and *Rasal3*^*−/−*^ mice were significantly shorter than B6 controls (Fig. 4b, c). Histological examination of colon sections also showed a quantitatively higher pathology score in both *Arhgef2*^*−/−*^ and *Rasal3*^*−/−*^ mice compared to B6, based on criteria that included inflammatory cell infiltration, submucosal edema and surface epithelial degeneration (Fig. 4d). Increased cellular infiltration of cells in inflamed colons of *Arhgef2*^*−/−*^ and *Rasal3*^*−/−*^ mice (seen in the histological pathology scoring) was concomitant to increased expression of *Ccdc88b* mRNA (Fig. 4e), a marker of myeloid and lymphoid cells^13^. Furthermore, expression of mRNAs for pro-inflammatory cytokines (IL6), chemoattractant (MCP1, RANTES), markers of cellular infiltration (MGL1, F4/80), and C4b, were significantly elevated in the colon of DSS-treated *Rasal3*^*−/−*^ and, to a lesser degree in *Arhgef2*^*−/−*^ mice compared to B6 (Fig. 4f). Lastly, immunohistological studies of colon sections revealed that both *Rasal3*^*−/−*^ and *Arhgef2*^*−/−*^ DSS-treated mice show reduced level of mucin (Fig. 4g, h). Quantification of cellular infiltrates (2 distalmost fields of view of each colon) revealed a significantly increased infiltration of CD3^+^ T cells and CD68^+^ mononuclear phagocytes, as well as a decreased number of KI-67^+^ proliferating cells in DSS-treated *Rasal3*^*−/−*^ mice compared to B6, all in agreement with greater susceptibility in *Rasal3*^*−/−*^ mice (Fig. 4g, h). Interestingly, cellular infiltrates in the colons of DSS-treated *Arhgef2*^*−/−*^ mice were quantitatively similar to those seen in B6 controls (Fig. 4g, h), and this despite a stronger inflammatory environment (Fig. 4f), suggesting possible differences in the mechanistic basis of DSS-susceptibility in *Rasal3*^*−/−*^ and *Arhgef2*^*−/−*^ mutants.

To further investigate the contribution of lymphoid cells to the exacerbated colitis phenotype seen in *Rasal3*^*−/−*^ and *Arhgef2*^*−/−*^ mice, we tested the ability of B6, *Rasal3*^*−/−*^ and *Arhgef2*^*−/−*^ naïve CD4^+^ CD25^−^CD45RB^Hi^ T cells to induce colitis upon adaptive transfer into lymphopenic *Rag1*^*−/−*^ mice. Seven weeks post-transfer, histology of colon sections revealed no difference between B6 and *Arhgef2*^*−/−*^, including a similar CD3^+^ T cells infiltration (Supplementary Fig. 4a) and pathology score (Supplementary Fig. 4b), suggesting that the exacerbated colitis response to DSS seen in *Arhgef2*^*−/−*^ mice (Fig. 4) was not lymphoid in nature. Conversely, transfer of *Rasal3*^*−/−*^ naive T cells failed to induce colitis when compared to B6, exhibiting both a decrease in the amount of infiltrating CD3^+^ T cells (Supplementary Fig. 4a) and a lower pathology score (Supplementary Fig. 4b). This suggests that RASAL3 is require for the proper function of T lymphocytes during colitis, in agreement with recently published studies^25,26^.

**Fig. 5:**
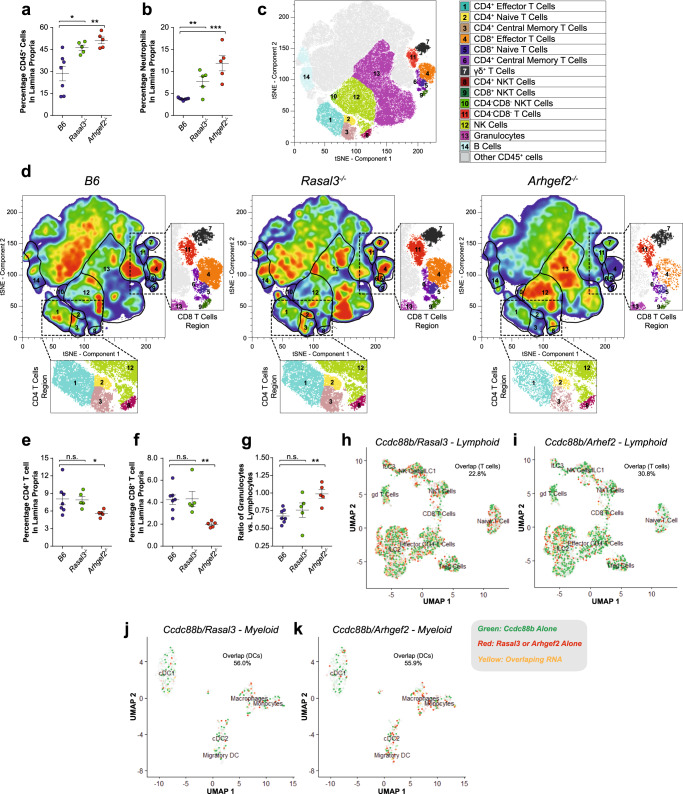
Cellular infiltrate in the lamina propria of *RASAL3*^*−/−*^ and *ARHGEF2*^*−/−*^ mice following DSS-induced colitis. Control B6, as well as *Rasal3*^*−/−*^ and *Arhgef2*^*−/−*^ mutant mice were given 3% DSS for 5 days followed by 3 days of water and were then sacrificed. **a**, **b** Percentage of total CD45^+^ cells and neutrophils in the lamina propria from B6, *Rasal3*^*−/−*^ and *Arhgef2*^*−/−*^ mice colon at day 8 following DSS treatment, respectively (mean ± SEM; n.s. non-significant, *p < 0.05, **p < 0.01, ***p < 0.001; two-tailed Student’s *t* test). **c** tSNE-based visualization of the different CD45^+^ cell populations found in the lamina propria, colored and numbered as per the legend (pooled analysis from all genotypes). **d** Same visualization as in (**c**), but for B6 (left), *Rasal3*^*−/−*^ (middle) and *Arhgef2*^*−/−*^ (right) mice colon at day 8 following DSS treatment, with the highest cellular density in red. Inserts depict regions of the tSNE plots corresponding to the CD8 and CD4 T cells clusters. **e**, **f** Percentage of CD4^+^ and CD8^**+**^ T cells from (**d**), respectively (mean ± SEM; *p < 0.05, **p < 0.01; two-tailed Student’s *t* test). **g** Ratio of total number of granulocytes vs. total number of lymphocytes from (**d**) (mean ± SEM; n.s. non-significant, **p < 0.01; two-tailed Student’s *t* test). tSNE-based visualization of single-cell RNAseq data from B6 lamina propria cells obtained at steady state for indicated lymphoid (**h**, **i**) and myeloid (**j**, **k**) populations. Expression of *Ccdc88b* is indicated in green and level of expression of either *Rasal3* (**h**, **j**) or *Arhgef2* (**i**, **k**) in red, as indicated in the legend. Indicated percentages represent the fraction of cells co-expressing RNA for *Rasal3* or *Arhgef2* with *Ccdc88b*, for the indicated populations.

To better understand the susceptibility of *Arhgef2*^*−/−*^ and *Rasal3*^*−/−*^ mice to colitis, we conducted cellular immunophenotyping of colon cell populations by flow cytometry following DSS treatment. These showed a greater infiltration of CD45^+^ cells in lamina propria of *Arhgef2*^*−/−*^ and *Rasal3*^*−/−*^ mice (Fig. 5a), with an important contribution from CD11b^+^/Ly6G^+^ neutrophils in both mutants compared to controls (Fig. 5b). Moreover, unsupervised tSNE visualization in *Arhgef2*^*−/−*^ mice revealed a significantly reduced proportion and number of both CD4^+^ and CD8^+^ T cells (populations 1 to 6, Fig. 5c, d) in this infiltrate compared to B6 and *Rasal3*^*−/−*^ mice (Fig. 5e, f) This abnormal cellular infiltration leads to a higher granulocytes to lymphocytes ratio in *Arhgef2*^*−/−*^ mice (Fig. 5g), suggesting further gene-specific effects of the mutation on the type of cellular infiltrate at the site of inflammation/tissue damage. Taken together, these results indicate an altered response of *Rasal3*^*−/−*^ and *Arhgef2*^*−/−*^ mice to acute DSS-induced colitis, with higher susceptibility and increased pathology and enhanced inflammatory response. Furthermore, we note unique differences in the composition of the cellular infiltrates in the gut of *Arhgef2*^*−/−*^ mutant mice. These results point to both a requirement for RASAL3 and ARHGEF2 in neuroinflammation (in the EAE model), while their absence is associated with increased inflammation in the gut following DSS-induced colitis.

We have previously observed that in other single gene models of immunodeficiency, dysregulated inflammatory response causes not only protection against acute neuroinflammation but also causes increased susceptibility to colitis-associated colorectal cancer (CA-CRC)^27^. Hence, we tested the impact of loss of function for *Rasal3* and *Arhgef2* on response to CA-CRC induced by combined treatment with DSS and the carcinogen azoxymethane (AOM). We note that as opposed to *Arhgef2*^*−/−*^ mice that behaved like controls, *Rasal3*^*−/−*^ mice showed a significantly greater susceptibility to CA-CRC; this susceptibility was expressed both by an increase in the total number of tumors per colon (Supplementary Fig. 5a, b), and an increase total tumor surface area (Supplementary Fig. 5c). Immunohistological studies of colon sections also revealed an exacerbated response in *Rasal3*^*−/−*^ mice (as shown by H&E and pSTAT3 staining), with a higher cellular proliferation (identified by KI67^+^ cells) and greater infiltration of CD3^+^ T cells (Supplementary Fig. 5d). Overall, *Rasal3*^*−/−*^ mice display an enhanced susceptibility to AOM DSS-induced CA-CRC model when compared to B6, with an increase cellular proliferation and T cell infiltration in the gut which is not seen in *Arhgef2*^*−/−*^ mice.

Finally, to explore the co-expression of *Ccdc88b* with *Rasal3* or *Arhgef2* mRNAs in colon immune cells we performed single-cell RNA sequencing at steady state. In lymphoid cells (Fig. 5h, i), a large fraction of T cells expressing *Ccdc88b* also express *Rasal3* (22.8% of *Ccdc88b*^+^ cells, Fig. 5h) and *Arhgef2* (30.8% of *Ccdc88b*^*+*^ cells, Fig. 5i). In myeloid cells, an even larger proportion of cells that express *Ccdc88b* also express *Rasal3* (56.0% of *Ccdc88b*^*+*^ cells, Fig. 5j) and *Arhgef2* (55.9% of *Ccdc88b*^*+*^ cells, Fig. 5k). Co-expression of both *Ccdc88b* with *Rasal3* and *Arhgef2* in DCs is of particular interest, given that CCDC88B expression was shown to be essential for the mobility and inflammatory function of these cells, both in vitro and in vivo^10^, raising the possibility that RASAL3 and ARHGEF2 may also be involved in migratory properties of these cells.

### Rasal3^−/−^ and Arhgef2^−/−^ dendritic cells exhibit altered mobility in vivo

**Fig. 6:**
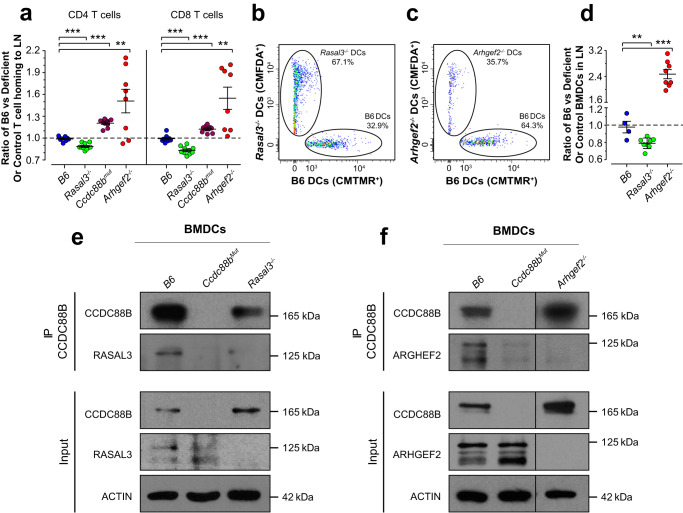
RASAL3 and ARHGEF2 are required for migration of dendritic cells in vivo. **a** Total spleen cells from control B6 and either *Ccdc88b*^*mut*^, *Rasal3*^*−/−*^ or *Arhgef2*^*−/−*^ mutants were stained with CMFDA or CMTMR dye, mixed at a 1:1 ratio and injected intravenously into B6 mice. Inguinal lymph nodes (LNs) were harvested 6 h later and analyzed by flow cytometry for number of CD4^+^ or CD8^+^ T cells. Numbers are expressed as a fold enrichment of dyed B6 T cells over dyed cells from *Ccdc88b*^*mut*^, *Rasal3*^*−/−*^, *Arhgef2*^*−/−*^ mutants or control B6 (means ± SEM, **p < 0.01, ***p < 0.001; two-tailed Student’s *t* test). **b**, **c** Control B6 and either *Rasal3*^*−/−*^ or *Arhgef2*^*−/−*^ LPS pulsed BMDCs were stained with CMFDA or CMTMR dye, mixed together at a 1:1 ratio and injected sub-cutaneously in the footpad of B6 mice. Draining popliteal LNs were harvested 48 h later and analyzed by flow cytometry (plot gated on CMFDA^+^ and CMTMR^+^ dyed cells). **d** Quantification from (**b**) and (**c**), expressed as a fold enrichment of dyed B6 BMDCs over dyed *Rasal3*^*−/−*^, *Arhgef2*^*−/−*^ or control B6 BMDCs (means ± SEM, **p < 0.01, ***p < 0.001; two-tailed Student *t* test). Data from (**a**) to (**d**) are representative of three independent experiments. **e**, **f** Lysates from B6, *Ccdc88b*^*Mut*^, *Rasal3*^*−/−*^ and *Arhgef2*^*−/−*^ BMDCs were immunoprecipitated with an anti^−^CCDC88B polyclonal anti-serum, and RASAL3 or ARHGEF2 proteins were detected by immunoblotting. Data from (**e**) and (**f**) are representative of two independent experiments.

We have shown that CCDC88B is required for mobility of myeloid (DCs) and lymphoid cells in vivo^10^. We thus assessed the loss of *Rasal3*^*−/−*^ and *Arhgef2*^*−/−*^ function on mobility of T cells (Fig. 6a). These studies revealed that CD4^+^ and CD8^+^ T cells from *Ccdc88b*^*mut*^ and *Arhgef2*^*−/−*^ mutants showed a reduced capacity to home to the inguinal LN. Surprisingly, CD4^+^ and CD8^+^ T cells from *Rasal3*^*−/−*^ showed a greater homing capacity than B6 cells in this model (Fig. 6a). Overall, these results suggest that a functional CCDC88B/ARHGEF2/RASAL3 complex is required for mobility of T lymphocytes in vivo.

We also assessed the role of ARHGEF2 and RASAL3 in migration of DCs in vivo. For this, primary BMDCs from B6 controls and from *Rasal3*^*−/−*^ or *Arhgef2*^*−/−*^ mutant mice were pulsed with LPS in vitro, followed by labeling with different fluorescent dyes (Fig. 6b, c). The ratio of labeled B6 to *Rasal3*^*−/−*^ DCs was found to be significantly reduced, suggesting enhanced migration of *Rasal3*^*−/−*^ DCs compared to B6 (Fig. 6b, d). Conversely, the ratio of B6 to *Arhgef2*^*−/−*^ DCs was found to be significantly higher, in agreement with decreased migration of *Arhgef2*^*−/−*^ DCs (Fig. 6c, d), similar to what we previously reported for *Ccdc88b*^*mut*^ DCs^10^. Taken together, these results indicate that similarly to CCDC88B, both ARHGEF2 and RASAL3 are required for proper migration of DCs in vivo.

Next, given that RASAL3, ARHGEF2 and CCDC88B all play a role in DCs migration and that they do physically interact in T lymphocytes, we evaluated if these proteins also interact together in DCs. Immunoprecipitation of CCDC88B in BMDCs extracts from the B6, *Ccdc88b*^*Mut*^, *Rasal3*^*−/−*^ and *Arhgef2*^*−/−*^showed that both RASAL3 (Fig. 6e) and ARHGEF2 (Fig. 6f) were pulled down, indicating that both proteins interact with CCDC88B in DCs. A similar immunoprecipitation using *Ccdc88b*^*Mut*^ DCs did not pull down either protein (Fig. 6e, f and Supplementary Fig. 6a, b), demonstrating the specificity of the interaction. These results show that the CCDC88B/ARHGEF2/RASAL3 complexes, initially identified in thymocytes and T cells, are required for proper migration of DCs and T cells.

### Rasal3 and Arhgef2 modulate dendritic cells motility in vitro through RHOA activation

**Fig. 7:**
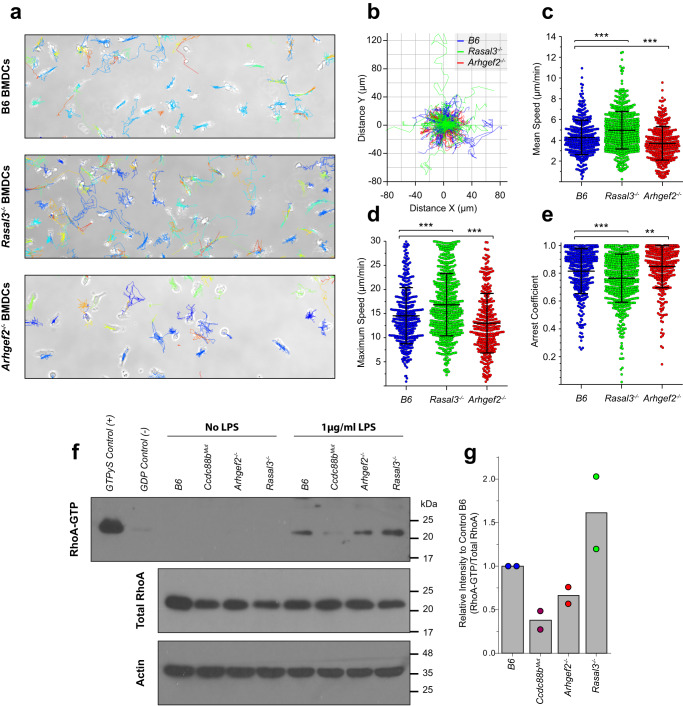
Altered motility parameters of *Rasal3*^*−/−*^ and *Arhgef2*^*−/−*^ dendritic cells. BMDCs generated from B6, *Rasal3*^*−/−*^ and *Arhgef2*^*−/−*^ mutants were imaged by bright-field time-lapse microscopy for 90 min and tracked using the TrackMate plugin from the ImageJ software, with manual correction. **a** Representative field of view of B6, *Rasal3*^*−/−*^ and *Arhgef2*^*−/−*^ BMDC cultures with the tracking results over the course of the experiment. **b** Normalized Rose plot depicting 25 randomly chosen track of individual B6 controls (blue), *Rasal3*^*−/−*^ (green) or *Arhgef2*^*−/−*^ (red) DCs. **c** Average velocity of individual BMDCs from control B6, *Rasal3*^*−/−*^ and *Arhgef2*^*−/−*^ DCs mutants (means ± SD, ***p < 0.001; two-tailed Student’s *t* test). **d** Maximum speed reached by individual DCs (means ± SD, ***p < 0.001; two-tailed Student’s *t* test). **e** Arrest coefficient using a threshold of 7 µm/min for actively patrolling DCs (means ± SD, **p < 0.01, ***p < 0.001; two-tailed Student’s *t* test). Data from (**a**) to (**e**) were processed using a homemade MatLab script and are representative of three independent experiments, taking at least two separated field of view (0.314 mm^2^ per field of view) per DCs culture. **f** BMDCs from B6, *Ccdc88b*^*Mut*^, *Rasal3*^*−/−*^ and *Arhgef2*^*−/−*^ mutants were stimulated with LPS and protein lysate analyzed for the level of active RhoA-GTP using Rhotekin-RBD (Rho Binding Domain) beads. As control, some BMDCs were loaded with high concentration of non-hydolyzable GTP (GTPγS, positive control) or GDP (negative control) prior to pull-down. **g** Quantification of (**f**), normalized to the total amount of RhoA expression, for two independent experiments.

We next investigated whether the physical interaction detected between CCDC88B, RASAL3 and ARHGEF2 in DCs translates into functional interaction at the cellular level, including a possible role in DC cells mobility in vitro. For this, we used an in vitro patrolling assay at steady state (Fig. 7a); similar to the reduced mobility phenotype previously observed in *Ccdc88b*^*Mut*^ DCs^10^, *Arhgef2*^*−/−*^ DCs were found to travel a significantly shorter distance than control DCs (Fig. 7a, b). Conversely, *Rasal3*^*−/−*^ DCs displayed the opposite phenotype with an increased traveled distance compared to controls (Fig. 7a, b). This was further reflected by the higher mean speed and maximum speed of *Rasal3*^*−/−*^ DCs (5.0 ± 1.8 µm/min and 16.9 ± 6.5 µm/min, respectively) when compared to B6 DCs (4.3 ± 1.7 µm/min and 14.6 ± 5.8 µm/min, respectively); contrariwise, the mean and maximum speeds of *Arhgef2*^*−/−*^ DCs were significantly slower (3.7 ± 1.6 µm/min and 13.0 ± 6.2 µm/min, respectively) (Fig. 7c, d). Likewise, the arrest coefficient, calculated as the fraction of time that the cell does not move using a threshold of 7 µm/min, was respectively reduced for *Rasal3*^*−/−*^ DCs (0.76 ± 0.17 and increased for *Arhgef2*^*−/−*^ DCs (0.85 ± 0.15), when compared to B6 control cells (0.82 ± 0.16) (Fig. 7e).

ARHGEF2 is known to be required for the regulation of the small GTPase RHOA, which is itself a master regulator of cytoskeleton rearrangement and proper cellular movement^28^. Thus, we next tested the activation status of RHOA-GTP in B6, *Ccdc88b*^*Mut*^, *Rasal3*^*−/−*^ and *Arhgef2*^*−/−*^ DCs following stimulation with LPS, using Rhotekin-coupled beads (Fig. 7f and Supplementary Fig. 6c). Stimulation of B6 DCs with LPS leads to a rapid and strong activation of RHOA, indicated by the high level of RHOA-GTP (Fig. 7f). On the other hand, absence of ARHGEF2 leads to decrease level of activation of RHOA in DCs (Fig. 7f, g). Furthermore, *Ccdc88b*^*Mut*^ DCs completely failed to activate RHOA (Fig. 7f, g). Lastly, and in opposition to *Arhgef2*^*−/−*^ DCs, *Rasal3*^*−/−*^ DCs exhibited an over-activation of RHOA, with a higher level of RHOA-GTP than B6 control (Fig. 7f, g). These results indicate that while CCDC88B is required for the activation of RHOA, proper level of activation is also regulated by ARHGEF2 and, inversely, RASAL3.

Considering our previous demonstration of the requirement of CCDC88B for DC cells mobility^10^, our results strongly suggest that CCDC88B, RASAL3 and ARHGEF2 form part of a multi-protein complex that plays an important role in DCs movement, where CCDC88B acts as a protein scaffold for the recruitment of ARHGEF2 and RASAL3 to the proper localization where they are required to modulate the motility of DCs and T-cells. In agreement with our in vivo data, the opposite effect of inactivation of *Rasal3* and *Arhgef2* on cell mobility raises the possibility that the 2 proteins play an opposite functional or regulatory role in the biochemical activity of this complex. This model is further supported by biochemical data showing that absence of ARHGEF2 (or CCDC88B) leads to reduced RHOA activation (possibly through loss of GTPase activity of ARHGEF2), while absence of RASAL3 leads to over-activation of RHOA (possibly through absence of the RasGAP activity of RASAL3).

## Discussion

CCDC88B was discovered as a gene which inactivation protects against acute neuroinflammation in models of cerebral malaria^6^. Subsequently, we showed that (1) *Ccdc88b* inactivation also protects against inflammation in experimental colitis^13^, (2) *CCDC88B* is the human gene underlying the 11q13 locus associated with susceptibility to several inflammatory conditions, including IBD^6,13^, and (3) *CCDC88B* mRNA is regulated by cis-acting elements in human myeloid cells, and *CCDC88B* mRNA expression is positively correlated with disease risk in Crohn’s disease patients^6,13^. Subsequently, we showed that CCDC88B contributes to normal inflammatory response by regulating mobility and migration of myeloid cells (DCs) to the site of inflammation in vivo, and in vitro^10^. At the molecular level, the mechanism by which CCDC88B regulates leukocyte migration remains unknown.

Using a proteomics approach with CCDC88B-expressing primary thymocytes and a T-lymphocyte cell line, we identified ARHGEF2 and RASAL3 as CCDC88B interactors (Fig. 1). The physical interaction between CCDC88B and ARHGEF2/RASAL3 was validated by reciprocal co-immunoprecipitation and by double immunofluorescence (Fig. 2). Their functional interaction was established in vivo (Figs. 3 and 4): *Arhgef2*^*−/−*^ and *Rasal3*^*−/−*^ mutants display resistance to neuroinflammation in EAE (alike *Ccdc88b*^*mut*^ mice), and show altered inflammatory response during DSS induced colitis. Furthermore, *Ccdc88b*, *Rasal3*,and *Arhgef2* are co-expressed in T cells and DCs (Fig. 5). Finally, mutations in *Arhgef2* and *Rasal3* have a dramatic but opposite effect on the migratory properties of myeloid and lymphoid cells in vivo (Fig. 6) and in vitro (Fig. 7). These studies establish that CCDC88B, ARHGEF2 and RASAL3 are critical for leukocytes mobility and activation of immune and inflammatory responses.

Recent studies have established a close partnership between ARHGEF2 and the Rho family of small GTPases (RHOA) known to regulate contractibility and cell movements^29–32^. ARHGEF2 is a microtubule-associated RHOA-specific nucleotide exchange factor (stimulates conversion of RHOA-GDP to RHOA-GTP)^33^ that couples microtubule depolarization with Rho-mediated actin stress fiber formation and cell contraction^34,35^. The activation of ARHGEF2 in response to inflammatory, mitogenic, and morphogenic signals promotes activation of RHOA and stress fiber formation^34^. ARHGEF2 has been implicated in migratory function of fibroblasts, neutrophils, epithelial cells, and T lymphocytes^29–32,36^. In T cells, activation of ARHGEF2 by PPP2R2A is required for Th1/Th17 differentiation, and PPP2R2A deficiency in T cells dampens the ARHGEF2/RHOA/ROCK pathway activation, decreasing EAE pathogenesis^37^. In neutrophils, loss of ARHGEF2 function leads to reduced spreading, crawling, and migration in response to sheer stress^29^. More broadly, ARHGEF2-dependent regulation of cytoskeletal rearrangements have secondary effects on phagocytosis, intracellular pathogens recognition, interaction with bacterial effectors, response to viral RNAs, activation of IRF3, and production of type I interferon^38–40^. Finally, ARHGEF2 overexpression has been associated with tumor progression including hepatocellular carcinoma, high-grade melanomas and malignant megakaryocytes^34,41,42^. Disruption of ARHGEF2/RHOA signaling (removal of BNIP-2 scaffold) affects breast carcinoma cells migration^43^.

In agreement with this published work, we observed that ARHGEF2 forms part of a protein complex with CCDC88B that is required for migration and inflammatory functions of DCs. Indeed, *Arhgef2* mutant DCs show a migratory defect in vivo (homing assay) and show reduced mobility in vitro. *Arhgef2* mutants also show resistance to neuroinflammation in EAE^6,10^. Conversely, *Arhgef2* deficiency causes susceptibility to colitis, expressed as increased pathology score, increased production of inflammatory cytokines (IL6, MCP1, RANTES, C4b), increased infiltration of CD45^+^ cells in the lamina propria, with a marked abundance of CD11b^+^/Ly6G^+^ neutrophils. Colitis susceptibility in *Arhgef2* mutants is opposite to colitis resistance seen in *Ccdc88b* mutants^13^. The reason for this seemingly contradictory result can only be speculated on at the moment, but could involve different mutation-specific effects on cellular responses at the site of inflammation, with cellular infiltrates of different leukocytes composition including strong granulocytes content in *Arhgef2* vs. *Ccdc88b*. The compensatory granulocytic response during colitis in *Arhgef2*^*−/−*^ mice is similar to other mouse mutants with DC-deficiency, including *Irf1* mutants^27^.

**Fig. 8:**
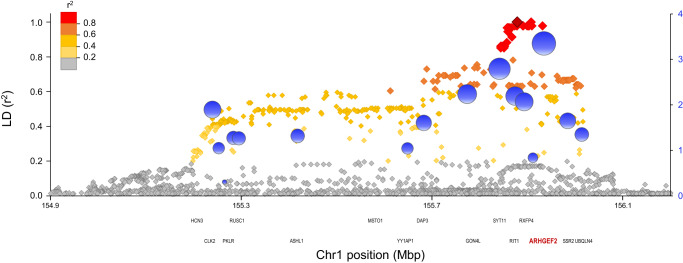
*ARHGEF2* containing IBD risk locus on chromosome 1 (1q22). The single nucleotide polymorphism (SNP) rs670523 was previously associated to IBD by GWAS^44^. The graph shows the linkage disequilibrium structure of the proxy SNPs to rs670523 (dark gray) calculated using LDlink^64^ as *r*^2^ values based on the 1000 Genomes phase 3v5 CEU + GBR European reference haplotypes (left *y* axis). The proxy SNPs are color-coded according to their *r*^2^ values and positioned at their chromosomal location (*x*-axis) over the genes found at this locus. The right *y*-axis shows the results of the MIS (Myeloid Inflammatory Score) epigenetic scoring method that we previously described^6^ and designed to prioritize candidate genes involved in inflammatory processes in myeloid cells based on functional genomics information. The MIS for each gene is shown by a blue bubble over its transcriptional start site with a diameter proportional to the MIS. *ARHGEF2* is found in strong LD with rs670523 and has the highest MIS within this IBD risk locus.

Finally, a locus on 1q22 containing 16 genes in linkage disequilibrium is associated with susceptibility to IBD^44^. The reported top-ranking marker (SNP rs670523) in this interval maps close to and is in almost complete LD with SNPs within or proximal to *ARHGEF2* (*r*^2^ score >0.96). Annotation of this locus using a Myeloid Inflammation Score (MIS)^6,45^ shows that *Arhgef2* has the highest MIS (3.4) in the locus interval (Fig. 8). These analyses together strongly suggest that *ARHGEF2* is the morbid gene at 1q22 locus (position 155 Mb). Hence, CCDC88B (11q13) and AHRGEF2 (1q22) physically and functionally interact, and are genetically linked to IBD susceptibility in humans.

RASAL3, is a member of the family of RAS GTPase-activating proteins (GAP), which negatively regulates RAS signaling by stimulating hydrolysis of RAS-GTP to RAS-GDP^23^. It has been shown to negatively regulate Ras/Erk signaling by stimulating p21RAS and Rac2 GTPase activity^23^. *Rasal3*^*−/−*^ mutant show decreased T cells and NKT cells numbers, display increased Erk phosphorylation and reduced production of cytokines by NKT cells^46^. In T cells, RASAL3 may repress TCR-signaling (ERK phosphorylation) which is required for T cells survival in vivo^25^, and *Rasal3*^*−/−*^ mutants display dampened inflammatory allergic dermatitis^26^.

**Fig. 9:**
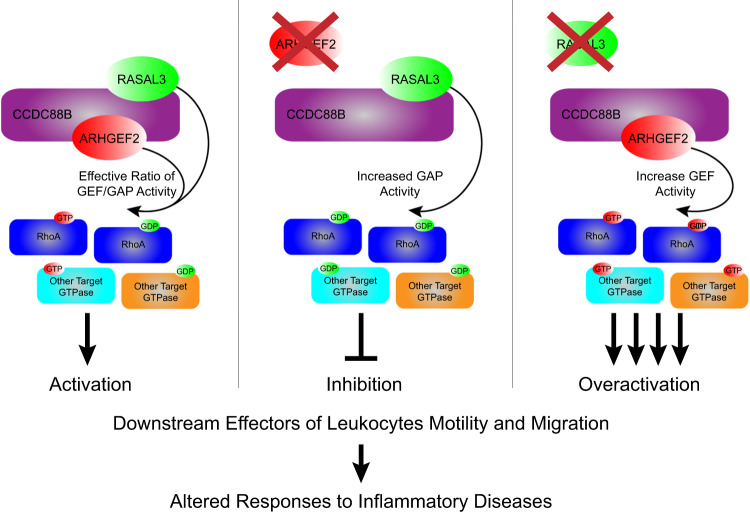
CCDC88B, ARHGEF2 and RASAL3 complex. Proposed interaction of CCDC88B with ARHGEF2 and RASAL3 in the context of cellular mobility (left), or in the absence of ARGHEF2 (middle) or RASAL3 (right), and their downstream target, RhoA. GEF guanine nucleotide exchange factor, GAP GTPase-activating protein.

Here, we show that RASAL3 forms a complex with CCDC88B and ARHGEF2 that plays an important role in the migration of DCs. *Rasal3*^*−/−*^ mutant DCs show both increased migration in vivo and enhanced mobility in vitro. This is opposite of *Ccdc88b* and *Arhgef2* mutant DCs measured in a similar way, suggesting that RASAL3 may act to negatively regulate cell migration in the context of the CCDC88B/RASAL3/ARHGEF2 complex (Fig. 9). These findings are similar to studies of FAM49B/CYRI^47,48^; CYRI binds to RAC1 in the GTP bound form and negatively regulates RAC1 GTPase activity. Inactivating CYRI mutations cause (1) increased mobility/migration of myeloid cells in vitro, (2) increased resistance to *Salmonella typhimurium*, (3) increased susceptibility to *Mycobacterium tuberculosis* and *Listeria monocytogenes*. It is tempting to speculate that RASAL3 may play a role functionally similar to CYRI in Rho/Rac signaling in regulating cell mobility.

*Rasal3* inactivation causes resistance to neuroinflammation in the ECM and EAE models (alike *Ccdc88b* and *Arhgef2* mutants) (Fig. 3). In addition, mutant *Rasal3*^*−/−*^ mice show enhanced susceptibility to DSS-colitis, which is similar to *Arhgef2* mutants (Fig. 4), including enhanced pathological scores, expression of inflammatory cytokine markers, and presence of CD45^+^ cellular infiltrate in the lamina propria and dominated by CD11b^+^/Ly6G^+^ granulocytes. Hence, although mutations in *Arhgef2* and *Rasal3* have opposite effects on DC migration, they cause similar pathological effects in DSS-colitis, albeit with different leukocytes infiltrates. Also, the *Rasal3* mutation causes a unique susceptibility to colitis-associated colorectal cancer phenotype not seen in the *Ccdc88b* or *Arhgef2* mutants (Supplementary Fig. 5). Such gene-specific effects could reflect unique function of the protein in T cells, DCs or other cells, or unique aspects of secondary response to inflammatory stimulus.

We propose a model where CCDC88B, ARHGEF2 and RASAL3 form a protein complex functionally critical for migration of lymphoid and myeloid cells. Although RASAL3 and ARHGEF2 are expressed in many cell types, CCDC88B is expressed exclusively in leukocytes possibly providing cell-specific function. Based in part on (1) the known but opposite regulatory role of ARHGEF2 and RASAL3 in Ras/Rho/Rac GTPase and associated signaling, (2) the known role of Rho/Rac GTPase in dynamic cytoskeletal structure, and function in cell migration, and (3) the phenotypic expression and inverse impact of loss of function at either *Arhgef2*, and *Rasal3* on DC cell mobility (Figs. 6 and 7), our findings suggest that ARHGEF2 and RASAL3 play an agonist and antagonist role in regulating the activity of this protein complex in cell movement by modulating the activation status of RHOA, and potentially other GTPases (Fig. 9). Finally, our findings in mouse models together with results from genetic association studies in humans for at least 2 of its components (*CCDC88B*, *ARHGEF2*) strongly suggest that this complex and genetic variants within its constituents may impact migration of inflammatory cells, and possibly impact genetic vulnerability to chronic inflammatory diseases in humans.

## Materials and methods

### Mice

Wild type C57BL/6J (B6) mice, 8–12 weeks of age, were obtained from the Jackson Laboratory (Bar Harbor, ME). Except were indicated, both male and female animals were used indiscriminately. The *Ccdc88b*^*m1PGrs*^ mutant mouse strain (referred to as *Ccdc88b*^*Mut*^) was generated by genome-wide chemical mutagenesis^6^. *Rasal3*^*−/−*^ mice were provided by Dr. H. Suzuki, from the National Center for Global Health and Medicine, Chiba (Japan), and is described elsewhere^25^. *Arhgef2*^*−/−*^ mice were generated by CRISPR/CAS9 by the Transgenic Core of the Life Sciences Complex at McGill University, using the experimental protocol and characterization strategy summarized in Supplementary Fig. 2. All mice were housed under specific pathogen-free conditions at the animal care facility of the Goodman Cancer Research Centre, McGill University, and the animal studies were conducted using protocols approved by the McGill Institutional Review Board (protocol number 5287) and following guidelines and regulations of the Canadian Council of Animal Care. We have complied with all relevant ethical regulations for animal use.

### Co-immunoprecipitation for SDS-PAGE and mass spectrometry analyses

Initial anti-CCDC88B co-immunoprecipitations from mouse primary thymocytes (Fig. 1a and Supplementary Data 1: MS/MS Expt #1) were carried out essentially as previously described^49^, using: (1) 200 mg of cryo-milled thymocytes; (2) 20 mM HEPES, pH 7.4, 0.5% (v/v) Triton X-100, 200 mM NaCl; and (3) an affinity-purified rabbit anti-CCDC88B polyclonal anti-serum^6^ coupled to Dynabeads M270 Epoxy in-house (adapted from ref. ^50^). This strategy was maintained for subsequent co-immunoprecipitations with changes as described herein. To prepare thymocytes, ~3 g of mice thymi were harvested, passed through a 70 µm cell strainer using a syringe plunger and washed with PBS. Cell suspensions were then centrifuged inside a syringe, the supernatant was removed, and the pellet was injected directly into liquid nitrogen. Two additional duplicate anti-CCDC88B co-immunoprecipitations from thymi were analyzed by liquid chromatography-tandem mass spectrometry (LC-MS/MS) analysis, prior to co-immunoprecipitation optimization screening. To further explore potential missing information in our IPs, an anti-CCDC88B co-immunoprecipitation based interaction screen^51^ was carried out on BI-141 T cells at ~50 mg per reaction, and the results are curated on www.copurification.org/. From this screen, three conditions were selected for further anti-CCDC88B co-immunoprecipitations, in triplicate, and label-free quantitative (LFQ) MS analysis from mouse primary thymocytes and BI-141 T cells: (1) 20 mM HEPES, pH 7.4, 200 mM NaCl, 0.5% (v/v) Triton X-100; (2) 20 mM HEPES, pH 7.4, 150 mM NaCl, 10 mM deoxy-Big CHAP; (3) 200 mM ammonium acetate, pH 7.0, 1% (v/v) Triton X-100. The criteria for selection of these conditions was based on visual inspection of the stained gel as follows: prominent staining of the band associated with CCDC88B and a relatively discrete pattern of other protein bands with some staining intensity differences between them.

### Sample workup for LC-MS/MS

Co-immunoprecipitated fractions were eluted in 1.1x NuPAGE sample loading buffer (Thermo Fisher Scientific #NP007) at 70 °C, reduced with DTT, alkylated with iodoacetamide, and run ~ 1 h at 200 V on a 1 mm 4–12% Bis-Tris NuPAGE gel followed by Sypro Ruby staining and protein band excision (Fig. 1a and Supplementary Data 1: MS/MS Expt #1) or as 4–6 mm gel-plugs, prior to Coomassie G-250 staining, peptide workup and LC-MS-MS (essentially as described in refs. ^52,53^). Gel bands or plugs were excised, cut into 1 mm cubes, de-stained, and digested overnight with 3.1 ng/μl trypsin (Promega, Madison, WI, #V5280) in 25 mM ammonium bicarbonate. Peptides were extracted from the gel in two incubations of 1 h each with 1.7% v/v formic acid, 67% v/v acetonitrile at room temperature with agitation. After partial evaporation by vacuum centrifugation to remove acetonitrile, digests were desalted on Stage Tips^54^. Stage Tip eluates were concentrated by vacuum centrifugation, loaded onto an Easy-Spray column (ES800, Thermo Fisher Scientific) and gradient-eluted (Solvent A = 0.1% v/v formic acid in water, Solvent B = 0.1% v/v formic acid in acetonitrile, flow rate 300 nl/min) into an Orbitrap Fusion Tribrid mass spectrometer (Thermo Fisher Scientific) acquiring data-dependent CID fragmentation spectra.

### MS data analysis

Files were submitted to MaxQuant^55^ for protein identification and LFQ (label-free quantification). Searches were performed against mouse protein sequences, exogenous contaminants, and a decoy database of reversed protein sequences. LFQ was performed separately for each of three buffer conditions. Non-mouse proteins and proteins that scored worse than any hit in the decoy database were removed from the MaxQuant output file “proteingroups.txt”. LFQ intensities were log2 transformed and samples were grouped by both cell type (thymocytes or BI-141 cells) and buffer condition. For each of the three immunoprecipitation conditions, respectively, proteins showing a LFQ intensity below three in at least one cell type were not considered. Missing values were imputed from a normal distribution with a down-shifted median relative to the measured data distribution, in order to simulate intensity near the detection limit. Triplicate immunoprecipitates from thymocytes and BI-141 cells in each immunoprecipitation condition were compared using two-sided Student’s *t* test as follow. Statistical significance was determined by a modified *t*-test that controls the relative importance of the Student’s *t* test *p* value and the fold change^56,57^ with a threshold for FDR (permutations) of 0.01. The RAW and MaxQuant processed files are available for download via ProteomeXchange with identifier PXD023779. Furthermore, a series of filters were used in a decision tree to prioritize possible CCDC88B interactors for further validation. First, candidate CCDC88B interactors were given priority if they were detected in at least 2 independent experiments, in different elution conditions, and if they were detected with immunoprecipitates from both primary thymus and BI-141 cells. Peptide coverage and abundance (peptide counts) in the extracts and relative intensity were also considered to prioritize interactors (Supplementary Fig. 1). Abundant proteins often found in co-immunoprecipitates (CRAPome v1.1) were excluded^22^. Since CCDC88B is known to be a cytoplasmic protein with microtubule interacting domains^6,13^, all nuclear proteins and DNA-binding proteins were also excluded from the analysis. A ponderation score was established (Supplementary Data 1 and Supplementary Fig. 1) based on these criteria and identified RASAL3 and ARHGEF2 as two prioritized CCDC88B interactors.

### Immunoprecipitation and immunoblotting

HEK293T cells were transiently transfected with a combination of expression plasmids encoding HA-CCDC88B^6^, RASAL3-FLAG (EX-Mm25984-M14, GeneCopoeia) or ARHGEF2-FLAG (EX-Mm19287-M14, GeneCopoeia) epitope tagged proteins, using the lipofectamine 2000 system (Thermo Fisher). Twenty-four hours later, transfected cell pellets were harvested and resuspended in RIPA lysis buffer (20 mM Tris-HCl, pH 7.5, 200 mM NaCl, 1 mM EDTA 1 mM EGTA 1% NP-40, 2.5 mM sodium pyrophosphate), supplemented with protease inhibitors (complete ultra protease inhibitor, Roche). Samples were clarified by centrifugation and incubated with monoclonal primary antibodies anti-FLAG (Sigma), or anti-HA (Santa Cruz Biotechnology), or IgG used as a negative control; immune complexes were recovered using protein G beads (Dynabeads). Beads were washed extensively, and protein eluted using boiling Laemmli buffer (2% SDS, 10% glycerol, 5% 2-mercaptoethanol) and analyzed by SDS-PAGE. In some experiments, bone marrow-derived dendritic cells (BMDCs) were generated from B6, *Ccdc88b*^*mut*^, *Rasal3*^*−/−*^, *Arhgef2*^*−/−*^ bone marrow as described previously^10^. These cells were lysed in 20 mM HEPES, pH7.4, 300 mM NaCl, 1% NP-40 (supplemented with protease inhibitors). Samples were clarified by centrifugation, pre-cleared and incubated overnight with affinity-purified rabbit anti-CCDC88B hyperimmune serum^6^; immune complexes were recovered using Sera-Mag Speedbeads Protein A/G Magnetic Particles (Sigma) according to the manufacturer protocol. For immunoblotting, proteins were separated by SDS-PAGE and transferred onto nitrocellulose membrane (GE-Healthcare), followed by incubation with rabbit anti-CCDC88B hyperimmune serum, followed by detection with HRP anti-rabbit antibodies (GE-Healthcare) and ECL, as previously described^6^. In some experiments, an affinity-purified rabbit anti-RASAL3 hyperimmune serum^25^ or anti-ARHGEF2 antibody (Cell Signaling Technology) were used (both at 1:300 dilution). Unprocessed original Western Blot analyses can be found in Supplementary Fig. 6.

### Immunofluorescence

Transfected HEK293T cells expressing HA-CCDC88B, in combination with either RASAL3-FLAG or ARHGEF2-FLAG, were fixed for 5 min with methanol at −20 °C, followed by permeabilization for 4 min at −20 °C with 30% methanol/70% acetone. Fixed cells were blocked with 3% BSA in PBS for 20 min and incubated overnight with the primary antibody anti-CCDC88B (rabbit polyclonal^6^, 1:200) or anti-HA (Santa Cruz Biotechnology, 1:1000) with either anti-RASAL3 (rabbit polyclonal, provided by Dr. Suzuki^25^, 1:200], anti-ARHGEF2 (Cell Signaling Technology, 1:200) or anti-FLAG (Sigma, 1:1000). Bound antibodies were detected using either Alexa488-coupled anti-rabbit IgG (Invitrogen) and Alexa594-coupled anti-mouse IgG (Invitrogen) secondary antibodies at a 1:000 dilution. In some experiments, cells were also counterstained with 4′,6-diamidino-2-phenylindole (DAPI, Invitrogen) to visualize the nuclei. Images were acquired using a Zeiss LSM710 Meta Laser Scanning Confocal microscope (100x lens) and processed with ImageJ (Fiji) with a maximum intensity projection.

### Plasmodium berghei ANKA Infection

Infections with *P. berghei ANKA* (PbA) were performed as we previously described^9^. Parasites were originally provided by the Malaria Reference and Research Reagent Resource Center (MR4) and were kept frozen at −80 °C in RPMI 1640 with 15% glycerol. Freshly thawed parasites were passaged once into B6 mice (7 days) and parasitemia in freshly harvested blood was monitored on stained thin blood smears (Diff-Quick reagents staining; Fisher Scientific) to prepare the infectious inoculum. PbA infection was performed using 10^6^ parasitized red blood cells (pRBCs) injected intravenously. Mice were then monitored for the appearance of neurological symptoms associated with cerebral malaria three times daily and euthanized when reaching clinical endpoints. All remaining animals were euthanized on day 19 post-infection (experimental endpoint).

### Experimental autoimmune encephalomyelitis

Experimental autoimmune encephalomyelitis (EAE) was induced as described elsewhere^9^. Briefly, female mice were injected subcutaneously with a short peptide of myelin oligodendrocyte glycoprotein (MOG, amino acids 35–55, 150 µg) emulsified in complete Freund’s adjuvant (50 μg/mouse). The same day and 2 days later, mice received 300 ng of pertussis toxin injected intraperitoneally. Mice were monitored daily for progressive paresis of tail and limbs; severely impaired animals were euthanized. Score was evaluated individually for the tail, each hind limb and each front limb on a scale from 0 to 3 (0, no symptoms; 1, weak; 2, full paresis; 3, no movement), for a possible maximum score of 10. The EAE incidence score was recorded as the highest score reached by each individual mouse over the course of the experiment. In some experiment, the mice were sacrificed at day 11, perfused, and the spinal cord harvested by flushing the spinal column. The spinal cord was then cut into small pieces and digested for 45 min in DNAseI (50 µg/ml, New England Biolabs) and collagenase V (1 mg/ml, Sigma). Single cells suspension was then prepared by passing through a 70 µm cell strainer using a syringe plunger and washed with PBS. Finally, the cells were stained with vital dye Zombie Aqua dye (1:400 dilution, Biolegend) then surface stained with the following fluorescently-labeled antibodies: 1:200 FITC anti-NK1.1 (clone PK136, eBioscience), 1:300 PerCP-Cy5.5 anti-Ly6G (clone 1A8, Biolegend), 1:300 APC anti-CD8α (clone 53-6.7, eBioscience), 1:400 APC-Fire 750 anti-CD45 (clone 30-F11, Biolegend), 1:1000 BV421 anti-Ly6C (clone HK1.4, Biolegend), 1:300 BV605 anti-CD11b (clone M1/70, Biolegend), 1:400 BV711 I-A/I-E anti-MHCII (clone M5/114.15.2, Biolegend), 1:300 BV785 anti-CD11c (clone N418, Biolegend), 1:400 PE anti-CD4 (clone RM4-5, Biolegend), 1:200 PE-Dazzle 594 anti-CD3 (clone 17A2, Biolegend) and 1:400 PE-Cy7 anti-F4/80 (clone BM8, Biolegend). Stained samples were processed using a Fortessa flow cytometer (BD Biosciences) and the results were analyzed using FlowJo software (Tree Star Inc). Gating strategy can be found in Supplementary Fig. 3, and each sample were normalized for 10 million living cells.

### Mouse model of intestinal colitis

The mouse model of DSS induced intestinal colitis was performed as we previously described^13^. Briefly, male mice were treated with 3% DSS (w/v, dextran sodium sulfate; MP Biomedicals) in their drinking water for 5 days, followed by 3 days of water. Mice were weighed daily throughout the experiments and euthanized at day 8. The entire colon was removed, measured and fixed in 10% neutral-buffered formalin overnight. Samples were embedded in paraffin, cut into 4 µm sections followed by staining with eosin and hematoxylin. To avoid bias, a trained pathologist scored each colon blindly on a scale of 36, with up to 4 points given in each of those categories: inflammatory cell infiltration, inflammatory cell depth, submucosal edema, increased mucosal thickening, surface epithelial degeneration, gland epithelial apoptosis, gland epithelial degeneration/abscesses, gland goblet/enterocyte ratio decrease and gland loss. In some experiments, colons from control and treated mice were instead dissociated with Lamina Propria Dissociation Kit (Miltenyi Biotec), according to the manufacturer instructions. Single-cell suspensions were prepared from colon, and stained with vital dye Zombie Aqua dye (1:400 dilution, Biolegend) then surface stained with the following fluorescently-labeled antibodies: 1:200 FITC anti-NK1.1 (clone PK136, eBioscience), 1:300 PerCP-Cy5.5 anti-CD4 (clone RM4-5, Biolegend), 1:200 PE anti-TCRγδ (clone GL3, eBioscience), 1:200 PE-Dazzle 594 anti-CD3 (clone 17A2, Biolegend), 1:200 PE-Cy7 anti-CD44 (clone IM7, eBioscience), 1:300 APC anti-CD62L (clone MEL-14, eBioscience), 1:200 AlexaFluor 700 anti-CD8α (clone 53-6.7, eBioscience), 1:400 APC-Fire 750 anti-CD45 (clone 30-F11, Biolegend) and 1:300 eFluor 450 anti-CD19 (clone eBio1D3, eBioscience). In some case, a myeloid panel was used instead: 1:300 FITC anti-CD64 (clone X54-5/7.1, Biolegend), 1:300 PerCP-Cy5.5 anti-Ly6G (clone 1A8, Biolegend), 1:200 PE anti-SiglecF (clone E50-2440, BDBioscience), 1:200 PE-Dazzle 594 anti-CD103 (clone 2E7, Biolegend), 1:400 PE-Cy7 anti-CD45 (30-F11, Biolegend), 1:200 APC anti-CD317 (clone 927, Biolegend), 1:800 APC-Cy7 I-A/I-E anti-MHCII (clone M5/114.15.2, Biolegend), 1:1000 BV421 anti-Ly6C (clone HK1.4, Biolegend), 1:300 BV605 anti-CD11b (clone M1/70, Biolegend), 1:300 BV785 anti-CD11c (clone N418, Biolegend) and 1:400 Zombie Aqua Fixable Viability (Biolegend). Stained samples were processed using a Fortessa flow cytometer (BD Biosciences) and the results were analyzed using FlowJo software (Tree Star Inc).

### Colon histology and immunochemistry

The fixed colon tissues were embedded in paraffin after dehydration, and 4 μm paraffin sections were prepared. After dewaxing and hydration, H&E staining was performed. The paraffin sections were scored as previously described^13^. To visualize goblet cells and mucin, an Alcian blue-PAS staining was performed. Sections were stained with Alcian blue at pH 2.5 (AB) followed by a staining step with the periodic acid-Schiff (PAS). For the IHC, paraffin-embedded tissue sections were de-waxed and rehydrated, incubated in Diva Decloaker antigen retrieval solution (Biocare) and boiled for 20 min in a pressure cooker; Enzyme Block (DAKO) was also used for 15 min to block peroxidase. Slides were stained with either 1:100 anti-Ki-67 (D3B5; Cell signaling), anti-CD68 (KP1, abcam), 1:250 anti-pSTAT3(Tyr705/D3A7, cell signaling), 1:100 anti-CD3 (SP7, Abcam) for 1 h. All antibodies were used in automated immunohistochemistry assays in accordance with the protocols recommended by the manufacturer (VENTANA BenchMark ULTRA instrument). Bound antibodies were detected using peroxidase-goat anti-rabbit secondary antibodies (AB_2307391, Jackson laboratories) used at a 1:500 dilution (VENTANA DISCOVERY series instruments). Slides were counter-stained with hematoxylin, then mounted. In some experiment, slides were then scanned and process using Aperio ImageScope (Version 12.4.3, Leica Biosystems Imaging Inc). The 2 most distal field of view (at 10x) were then systematically processed to quantify infiltration of immune cell populations using Fiji (Version 1.53f51^58^) using the following main operators: Thresholding (Otsu Binary), Automatic Smoothing/Sharpening, Watersheding and Analyze particle.

### Mouse model of T cell specific colitis

Colitis was induced as we previously described^13^. Briefly, spleen from B6, *Arhgef2*^*−/−*^ and *Rasal3*^*−/−*^ were enriched for naive CD4^+^ CD45RB^hi^ T cells (CD4^+^ T Cell Isolation Kit; Miltenyi Biotec) and single-cell suspensions was prepared. Cells were then then surface stained with the following fluorescently-labeled antibodies: 1:300 FITC anti-CD4 (GK1.5, eBioscience), 1:250 PE anti-CD25 (PC61.5,eBioscience) and 1:1500 APC anti-CD45RB (C363.16A, eBioscience). Naive cells where then purified by cell sorting (FACSAriaII, BD Biosciences) and 5 × 10^5^ CD4^+^ CD45RB^hi^ T cells were then injected intravenously into *Rag1*^*−/−*^ mice. Mice were sacrificed 7 weeks after injection, their colon harvested and processed as above.

### Gene expression and cytokine quantitation in colon tissues

RNA from distal colons flash-frozen in liquid nitrogen was prepared and analyzed as described elsewhere^13^. Briefly, RNA was purified using a FastPrep 24 homogenizer (MP Biomedicals) with lysing matrix D beads (MP Biomedicals) and RNeasy kits (QIAGEN). MMLV reverse transcriptase was used with a cocktail of Oligo dT and random hexamers (Invitrogen) for cDNA synthesis. The primer set (5′-CCGGGAGCTTCGAGGCCAAC-3′ and 5′-CCTATCTGGCAAGCGGGGC-3′) was used to quantify the relative expression of *Ccdc88b* mRNA. Cytokines and chemokines RNA transcripts were quantified by RT-qPCR using Perfecta SYBR Green PCR kit and the following primers sets: *Mcp*-1 (5′-AGGTGTCCCAAAGAAGCTGTA-3′ and 5′-TCTGGACCCATTCCTTCTTG-3′), *Il-6* (5′-GAAGTAGGGAAGGCCGTGG-3′ and 5′-GAAGTAGGGAAGGCCGTGG-3′), *Emr1* (F4/80) (5’-CTTTGGCTATGGGCTTCCAGTC-3’and 5’-GCAAGGAGGACAGAGTTTATCGTG-3), *Rantes* (5′-GCAAGTGCTCCAATCTTGCA-3′ and 5′-CTTCTCTGGGTTGGCACACA-3′). *Mgl1* (5’-GAGAAAGGCTTTAAGAACTGGG-3’ and 5’-GACCACCTGTAGTGATGTGGG-3’) and *C4b* (5’-GATGAGGTTCGCCTGCTATT-3’ and 5’-GACTTGGGTGATCTTGGACTC-3’) Gene expression was normalized to *Hprt* (5′-TCAGTCAACGGGGGACATAAA-3′ and 5′-GGGGCTGTACTGCTTAACCAG-3′) and relative expressions was calculated using the ΔΔCT method^59^.

### Mouse model of colitis-associated colorectal cancer

For the colitis-associated colorectal cancer model (CA-CRC), mice were injected intraperitoneally with azoxymethane (MP Biomedical; 7 mg/kg) followed by three 4-day cycles of 2% DSS in the drinking water, with each cycle 17 days apart. Mice were monitored at regular intervals and weighed weekly. Colons were collected after 14 weeks, fixed in formalin and analyzed for the number of tumors per colon, as well as their total tumor surface area (calculated from diameter of individual tumors). In some experiments, colon sections were also analyzed by immunohistochemistry as above.

### Single-cell RNA sequencing

Colons from B6 mice were collected, cut into small pieces, and washed in gut buffer (1X Hank’s Balanced Salt Solution (HBSS) containing 2% heat-inactivated fetal bovine serum (FBS) and 15 mM of HEPES. Epithelial cells were removed by shaking in gut buffer with 5 mM EDTA for 30 min at 37 °C, with occasional vortexing. Finally, tissues were treated with collagenase IV (20 mg/ml) and DNase I (10 mg/ml) in RPMI-1640 supplemented with 5% FBS and 15 mM of HEPES for 20 min at 37 °C and passed through a 70 μM cell strainer to collect lamina propria cells. Cells were stained for viability using Zombie Viability Dye V500 (1:400), and surface stained with anti-CD45 APC (1:400, clone 30F11, Biolegend). Stained cell suspensions were sorted on a BD FACSAriaIII Cell Sorter (BD Biosciences) to obtain viable CD45^+^ cells. Freshly sorted cells were washed and resuspended in 0.04% BSA in PBS for loading on the 10x Chromium chip. Single-cell capture and cDNA preparation was done according to the 10x Single-Cell 3’ (version 3.1) protocol, with 8000 cells targeted for capture per sample. Libraries were sequenced on the NovaSeq 6000 Sequencing System (Illumina). Raw sequencing data can be found on the Gene Expression Omnibus Repository (GEO)^60^ with the accession number GSE249342. Raw gene expression matrices were generated for each sample by the Cell Ranger, and the output was analyzed using the Seurat package. Low-quality reads were filtered based on three criteria: number of detected genes per cell, number of UMIs expressed per cell and mitochondrial content, using the following threshold parameters: nGene (between 200 and 7500), nUMI (between 500 and 75,000), and percentage of mitochondrial genes expressed (<7.5%). Doublets were then identified by finding cells expressing markers of two cell lineages simultaneously, as well as using the DoubletFinder package. After removal of low-quality cells and doublets, we captured 12,475 CD45^+^ cells from B6 colonic lamina propria (total from 2 samples). Gene expression matrices were normalized using the NormalizeData function and scaled using the ScaleData function, regressing out effects of cell cycle and percentage of expressed mitochondrial genes. Identification of highly variable features, linear dimension reduction by PCA transformation, UMAP dimensionality reduction, and cell clustering were performed using standard Seurat package workflows. Myeloid cells were integrated for further sub-clustering. Normalization, scaling, PCA, UMAP and clustering were performed as described above.

### T cell homing assay

Spleen were harvested, processed into single-cell suspensions and red blood cells lysed using ammonium chloride containing buffer (ACK; Fisher scientific). Splenocytes were stained with either 2 µM of CellTracker green (5-chloromethylfluorescein diacetate or CMFDA, ThermoFisher) or 20 µM of CellTracker orange (5-(and-6)-(((4-chloromethyl)benzoyl)amino)tetramethylrhodamine or CMTMR, Thermo Fisher) for 15 min at 37 °C. Stained cells were extensively washed with PBS, counted twice, and a mixture (1:1 ratio) of independently stained splenocytes (total of 1 × 10^7^ cells) were injected intravenously into the tail vein of B6 mice. Inguinal lymph nodes (LNs) were harvested 6 h later and processed for analysis of cell composition by flow cytometry using the following fluorescently-labeled antibodies in addition to CMFDA and CMTMR: 1:300 PerCP-Cy5.5 anti-CD4 (clone RM4-5, Biolegend), 1:400 APC anti-CD45 (clone 30-F11, eBioscience), 1:300 APC-Fire anti-CD8a (clone 53-6.7, eBioscience), 1:200 PE-Dazzle 594 anti-CD3 (clone 17A2, Biolegend) and Zombie Aqua Fixable Viability (Biolegend). The relative amount of CMFDA^+^ T cells compared to CMTMR^+^ T cells was calculated as follows: (% of CMFDA^+^ T cells in sample/% of CMFDA^+^ T cells in injection mix)/(% of CMTMR^+^ T cells in sample/% of CMTMR^+^ T cells in injection mix), as we described^10^.

### In vivo migration assay

Migration assays were performed as previously described^10^. LPS-treated bone marrow-derived dendritic cells (BMDC) were stained with either 2 µM of CellTracker green (CMFDA, Thermo Fisher) or 20 µM of CellTracker orange (CMTMR, Thermo Fisher) for 15 min at 37 °C, extensively washed with PBS, counted twice, and a mixture (1:1 ratio) of independently stained cells (total of 1 × 10^6^ cells) were injected in the footpad of B6 mice. Popliteal LNs were harvested 48 h later, and the ratio of CMFDA and CMTMR labeled CD11c^+^ cells was analyzed by flow cytometry. The relative amount of CMFDA^+^ DCs compared to CMTMR^+^ DCs was calculated as above.

### In vitro patrolling assay

Patrolling assays were performed as previously described^10^. BMDCs were cultured in Nunc Lab-Tek 8-wells cover-glass chambers (Thermo Fisher) for 3 h at 37 °C and 5% CO_2_ in RPMI supplemented with 10% fetal bovine serum (HyClone) before imaging. Two representative fields of view per sample were acquired every minute for 90 min by brightfield microscopy using a Zeiss LSM700. Images were processed with ImageJ (Fiji^58^) and analyzed using the built-in TrackMate plugin^61^ in addition to a homemade MatLab script (MathWorks). Single tracks produced by the TrackMate plugin were manually removed when tracking cellular debris or non-adherent cells.

### RHOA activation assay

Bone marrow-derived dendritic cells (BMDCs) were produced as described previously^10^, serum starved for 24 h and stimulated for 5 min at 37 °C in RPMI supplemented with 10% fetal bovine serum with 1 µg/ml LPS. BMDCs were then wash extensively with ice cold PBS and processed rapidly to a Rho Activation Assay Biochem Kit (Cytoskeleton Inc) accordingly to the manufacturer instructions. Briefly, BMDCs were lysed in 50 mM Tris pH 7.5, 10 mM MgCl2, 0.5 M NaCl, and 2% Igepal (Cytoskeleton Inc), and 800 µg of lysates submitted to immunoprecipitation with Rhotekin RBD beads IP for 1h15 at 4 °C with 50 µg beads. Lysate was then washed, eluted in 2x Laemmli sample buffer, boiled for 10 min and analyzed by SDS-PAGE and Western blot analysis using Anti-RHOA monoclonal IgM antibody (1:500 Cytoskeleton Inc), as described above. For this assay, positive control was also produced by saturating BMDCs lysate with 200 μM GTPγS and negative control was produced by saturating BMDCs with 1 mM GDP, before immunoprecipitation. Western blot images were scanned, processed with ImageJ (Fiji^58^) and analyzed for the relative intensity of RHOA-GTP when compared to total RHOA in the input.

### Statistical analysis and reproducibility

Results are presented as mean ± SEM, unless otherwise indicated. Prism5 (GraphPad) software was used for all statistical tests, using parametric tests when criteria for normality is satisfied and non-parametric otherwise, as indicated. Differences were considered statistically significant when *p* ≤ 0.05. *p* values are indicated by *p ≤ 0.05, **p < 0.01, ***p < 0.001. Data are independent biological replicate, where each individual data point in each plots represent a single mouse or primary cell line derived from an individual mouse, except when otherwise indicated; number of independent experiments carried out with similar results are identified in each corresponding Figure legend and were all reproducible.

### Reporting summary

Further information on research design is available in the Nature Portfolio Reporting Summary linked to this article.

### Supplementary information

Supplementary Information

Description of Supplementary Materials

Supplementary Data 1

Reporting Summary

## Data Availability

The MS proteomics data have been deposited to the ProteomeXchange Consortium via the PRIDE^62^ partner repository with the dataset PXD023779. The protein interactions from this publication have been submitted to the IMEx (http://www.imexconsortium.org) consortium through IntAct^63^ and assigned the identifier IM-28785. The raw single-cell RNA sequencing data can be found on the Gene Expression Omnibus Repository (GEO)^60^ with the accession code GSE249342. Any other data that support the findings of this study, including additional MS/MS data (see Supplementary Data 1), are available within the article, its Supplementary Information and from the corresponding author upon reasonable request.
