# Supplementary material for: CCDC88B interacts with RASAL3 and ARHGEF2 and regulates dendritic cell function in neuroinflammation and colitis
Source: Commun Biol. 2024 Jan 10;7:77. doi: 10.1038/s42003-023-05751-9 (PMC10781698; doi:10.1038/s42003-023-05751-9)
Supplement: Supplementary file 1 — Supplementary Information [file 42003_2023_5751_MOESM1_ESM.pdf]

```
> Rasal3, mouse, Q8C2K5
MKPECGQTMFRTFWSRSRDSSAMDPPLQSEEDSQTQPSLPSPLTSYRWHTGGSGEKAAGGFRWGRF
AGWGRALSHQEPMVNSQPAPRSLFRRVLSAPPKESRSNRLRFSKTLWGRHKNVAPLEPKPNPKAPE
PELELVADPDLPVAQIPEPPTPDMPVWNIDGFTLLEGKLVMLGEEEGPRQIRVGSASSENSMQAAL
GNLKDAVRTPGKTEPEAAGSNQVHNVRKLLKRLKEKKRAKSELGAYTPRDGPPSALGSRESLATLS
ELDLGAERDVRVWPLHPSLLGEPYCFQVTWAGGSLCFSCRSSAERDRWIEDLRRQFQPSQDNVERQ
EMWLTVWVHEAKGLPRATVPGVRAELWLDGALLARTAPRAGPGQLFWAERFHFEALPPARRLSLRL
RSAGPAGATVGRVVLELDEVSIPRAPAAGLERWFPVLGAPAGAVLRARIRVRCLRVLPSERYKELA
EFLTFHYARLCGALEPALSAQAKEELAAAMVRVLRATGRAQALVTDLGTAELARCGGREALLFREN
TLATKAIDEYMKLVAQEYLQDTLGQVVRCLCASTEDCEVDPSKCPTPELPKHQARLRDSCEEVFEN
IIHSYNCFPAELGSVFSSWREACKARGSEALGPRLVCASLFLRLLCPAILAPSLFGLAPEHPAPGP
ARTLTLIAKVIQNLANCAPFGEKEAYMAFMNSFLEDHGPAMQHFLDQVATVDADTTPSGYQGSGDL
ALQLAVLHVQLCTIFAELDQKTQDSLEPLPTILRAIEEGRPVPVSVPMRLPRISTQVQSSFFSGEK
PGFLAPRDLPKHTPLISKSQSLRSFQGAGSWASRRPDEERPQRRPRPVLRTQSVPARRPTHRRPSA
GSKPRPKGSLRMGPAPCGRAWTRASASLPRKPSVPWQRQMDQPGDRYQTTGTHRPVGKLAEIQCEV
AIFREAQKALSLLVESLSTQVQALKEQQEHFRCQLQDLYSRLGAGISKLDSKGGLPSNGSHRLKSL
EQRLTEMECSQDQLRDSLQSLQLLSKTPGSRSQPLPLKAPCVNGADLSMGT
```

Experiment #1
% Coverage = 38%
# Unique peptides = 49

Experiment #2
% Coverage = 56%
# Unique peptides = 55

```
> Arhgef2, mouse, Q60875
MSRIESLTRARIDRSKEQATKTREKEKMKEAKDARYTNGHLFTTISVSGMTMCYACNKSITAKEA
LICPTCNVTIHNRCKDTLANCTKVKQKQQKAALLRNNTALQSVSLRSKTTTRERPTSAIYPSDSF
RQSLLGSRRGLSSLSLAKSVSTTNIAGHFNDESPLGLRQILSQSTDSLNMRNRTLSVESLIDEGV
EVFYNELMSDFEMDEKDFEADSWSLAVDSSFLQQHKKEVMKKQDVIYELIQTELHHVRTLKIMTR
LFRTGMLEELQMEPEVVQGLFPCVDELSDIHTRFLNQLLERRRQALCPGSTRNFVIHRLGDLLIS
QFSGSNAEQMRKTYSEFCSRHTKALKLYKELYARDKRFQQFIRKMTRSAVLKRHGVQECILLVTQ
RITKYPVLINRILQNSHGVEEEYQDLASALGLVKELLSNVDQDVHELEKEARLQEIYNRMDPRAQ
TPVPGKGPFGRDELLRRKLIHEGCLLWKTATGRFKDVLLLLMTDVLVFLQEKDQKYIFTSLDKPS
VVSLQNLIVRDIANQAKGMFLISSGPPEMYEVHAASRDDRTTWIRVIQQSVRLCPSREDFPLIET
EDKAYLRRIKTKLQQKNQALVELLQKNVELFAEMVHFQALKAGFVGMPPPALPRGLFRLESFESL
RGERLLKDALREVEGLKDLLLGPCVDLPMTSREPALPLDSDSGSCPGVTANGEARTFNGSIELCR
ADSDSSQKDRNGNQLRSPQEEVLQPLINLYGLLHGLQAVVVQQERLMEALFPEGPERWEKLSRAN
SRDGEAGRAAVASVTPEKQATELALLQRQHTLLQEELRRCQRLGEERATEAGSLEARLRESEQAR
ALLEREAEEIRRQLAALGQNEPLPAEAPWARRPLDPRRRSLPAGDALYLSFNPPQPSRGHDRLDL
PVTVRSLHRPFDDREAQELGSPEDRLQDSSDPDTGSEEEVSSRLSPPHSPRDFTRMQDIPEETES
RDGEPTASES
```

Experiment #1
% Coverage = 40%
# Unique peptides = 45

Experiment #2
% Coverage = 45%
# Unique peptides = 38

Supplemental Figure 1. Protein Coverage and Unique Peptides Count of the Top Candidate CCDC88B Interactors. RASAL3 and ARHGEF2 were identified as the top CCDC88B candidate interactors by co-IP followed by LC-MS/MS as described in **Figure 1**. Their protein sequences are shown and the highlighted regions correspond to the sequence coverage by LC-MS/MS identified peptides from thymus and BI-141 T-cell line. The percentage of protein sequence coverage along with the number of unique peptides identified in two independent LC-MS/MS experiments are provided.

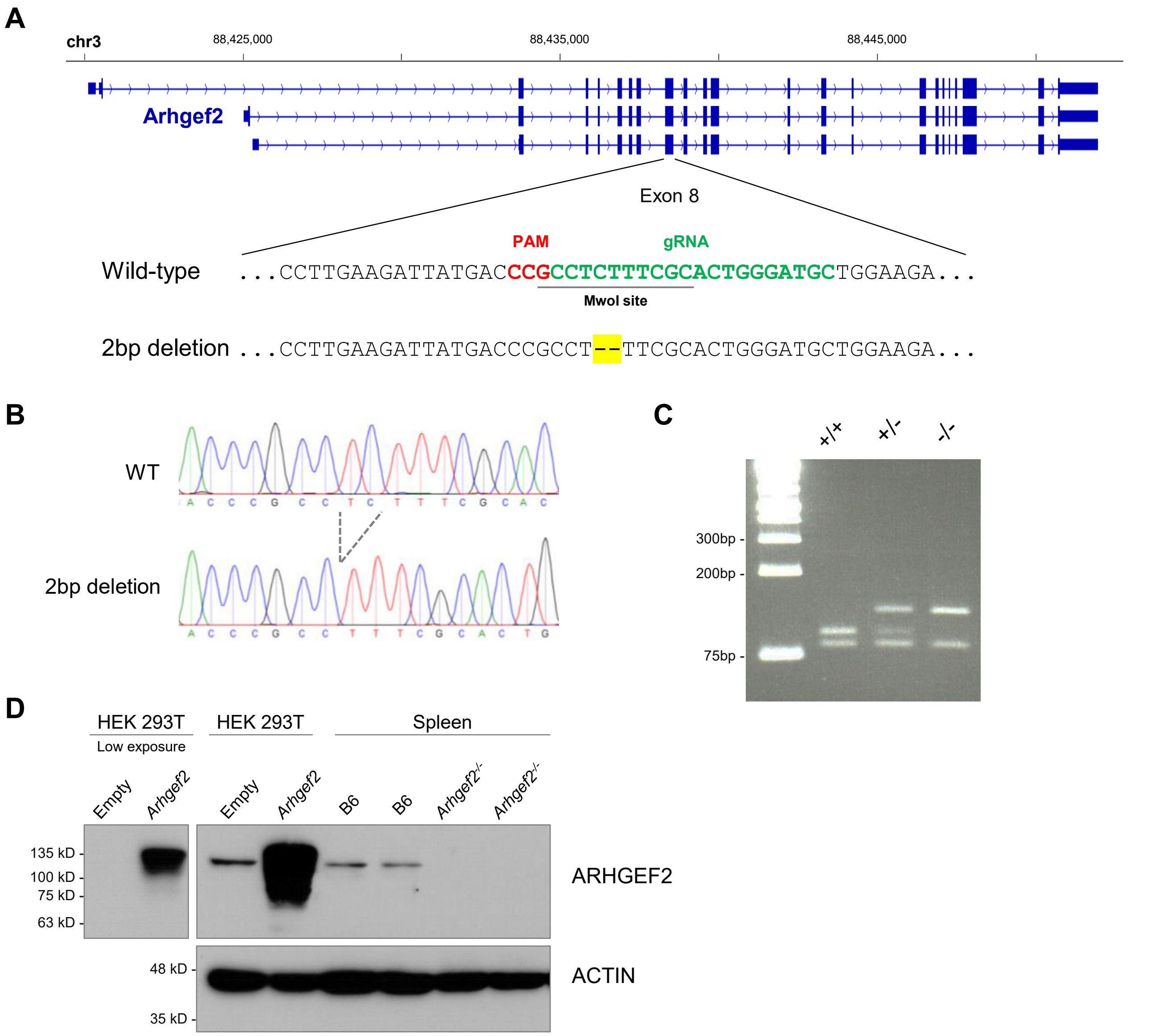

Supplemental Figure 2. Generation of *Arhgef2* Mutant Mice. (A) Strategy to generate the null allele at the *Arhgef2* gene. Cas9 cleavage guided by a sgRNA targeting exon 8 of *Arhgef2* created a two base pair deletion, bringing the coding sequence out of frame and creating an early stop codon. **(B)** Targeted sequencing profile showing the 2bp deletion in homozygous *Arhgef2* mutant mice. **(C)** The 2bp deletion abrogates a *MwoI* restriction site and is used for genotyping by PCR and digestion. **(D)** Western blot performed for ARHGEF2 and ACTIN on total protein extracts from spleen of two control B6 and *Arhgef2*$^{-/-}$ mutant mice. Protein extracts from HEK293T cells transfected with empty pcDNA3.1 or with expressing vector for mouse *Arhgef2*-3xFlag construct were analyzed by immunoblotting.

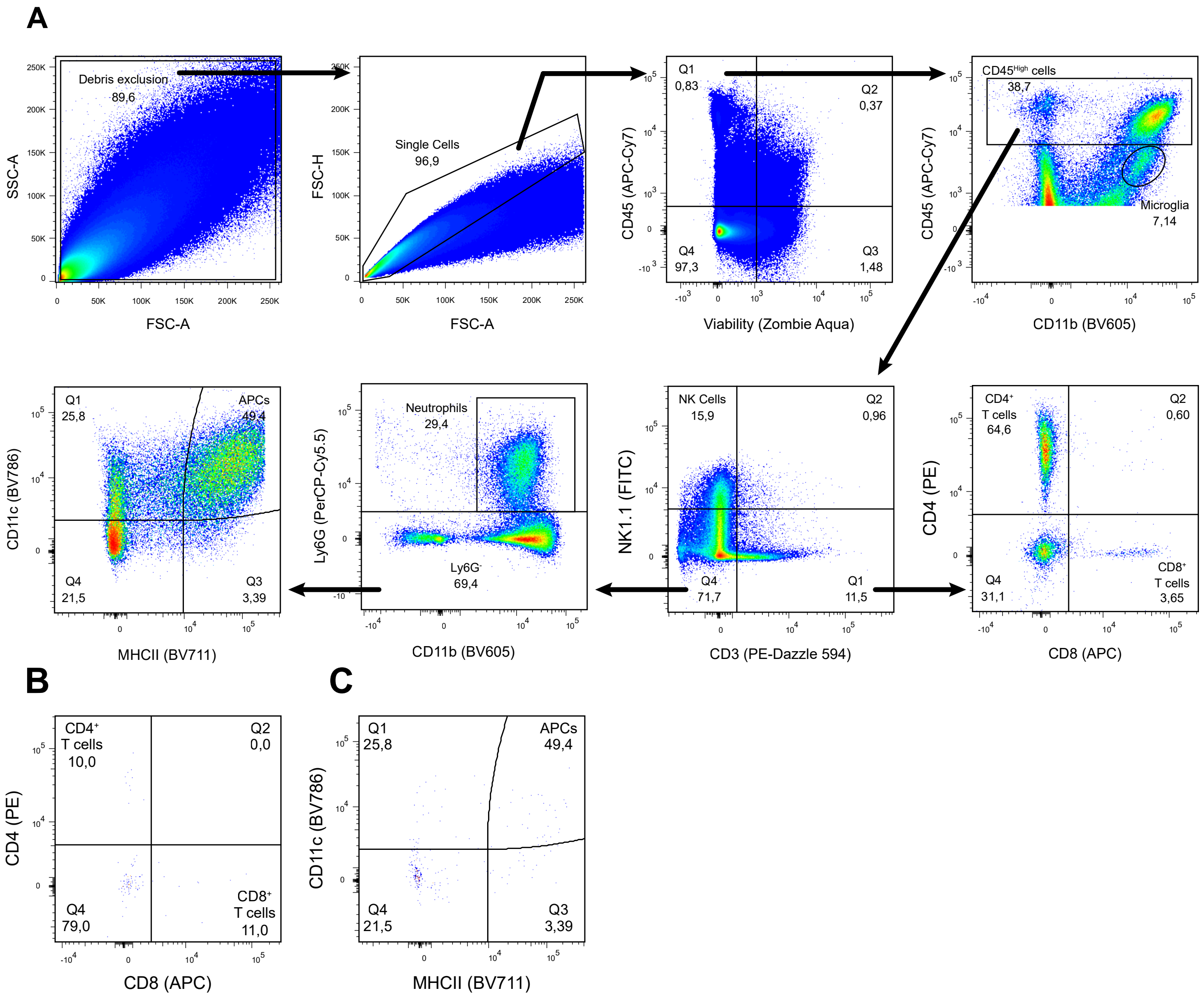

Supplemental Figure 3. Additional Flow Cytometry Data for Spinal Cord. (A) Gating strategy used in **Fig. 3G-3H** for spinal cord immune cells infiltration. **(B-C)** Flow cytometry for total $CD45^{+}CD3^{+}CD4^{+}$ T cells **(B)** and total $CD45^{+}CD11c^{High}MHCII^{High}$ antigen presenting cells **(C)** in control B6 spinal cord.

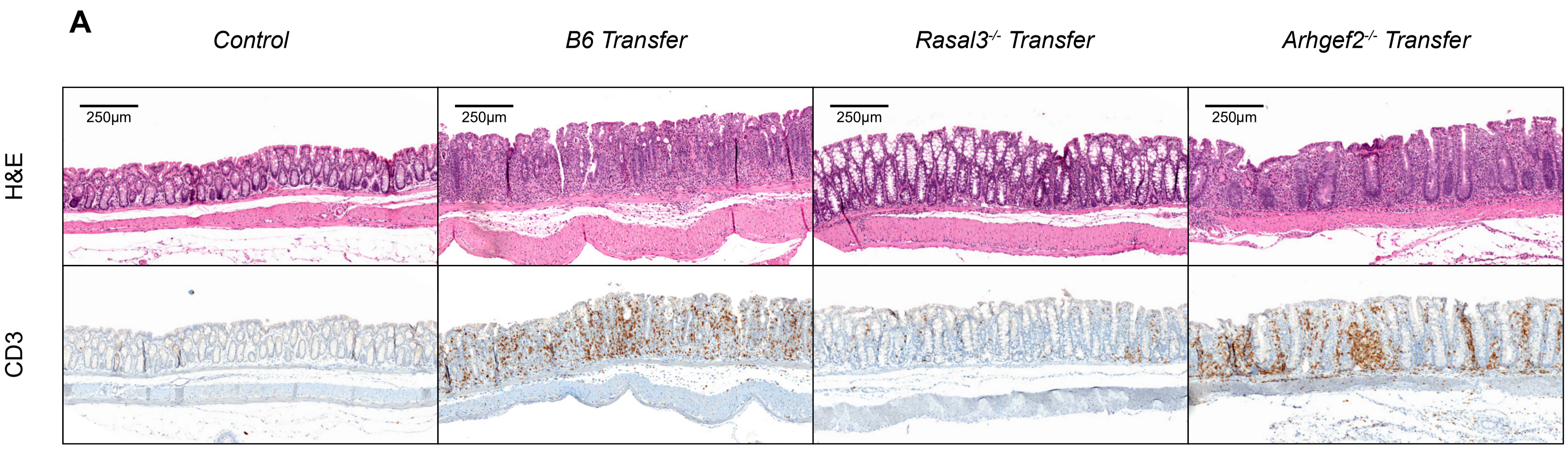

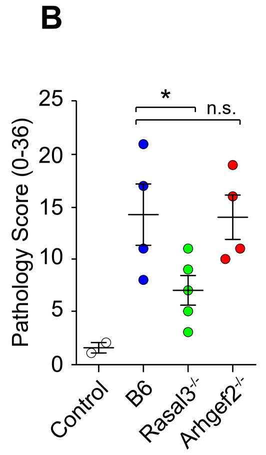

Supplemental Figure 4. *Rasal3$^{-/-}$* CD4^{+} T cells do not induce colitis in immunocompromised mice. (A-B) CD4^{+} CD25^{-}CD45RB^{Hi} T cells (4×10^5) from B6 or from *Rasal3$^{-/-}$* or *Arhgef2$^{-/-}$* mice were transferred into *Rag1$^{-/-}$* mice and colons were harvested 7 weeks later. **(A)** Hematoxylin and eosin staining (top) or immunohistochemistry staining for CD3 (bottom) of colon sections after transfer of B6, *Rasal3$^{-/-}$* and *Arhgef2$^{-/-}$* CD4^{+} T cells. Data are representative of at least 4 colons per group. Control: *Rag1$^{-/-}$* mice without T cells transfer **(B)** Pathology scores evaluating inflammatory cell infiltration, submucosal edema, gland loss and surface epithelial erosion/ulceration (mean ± SEM; n.s.: non-significant, *$P < 0.05$; two-tailed Student's t-test).

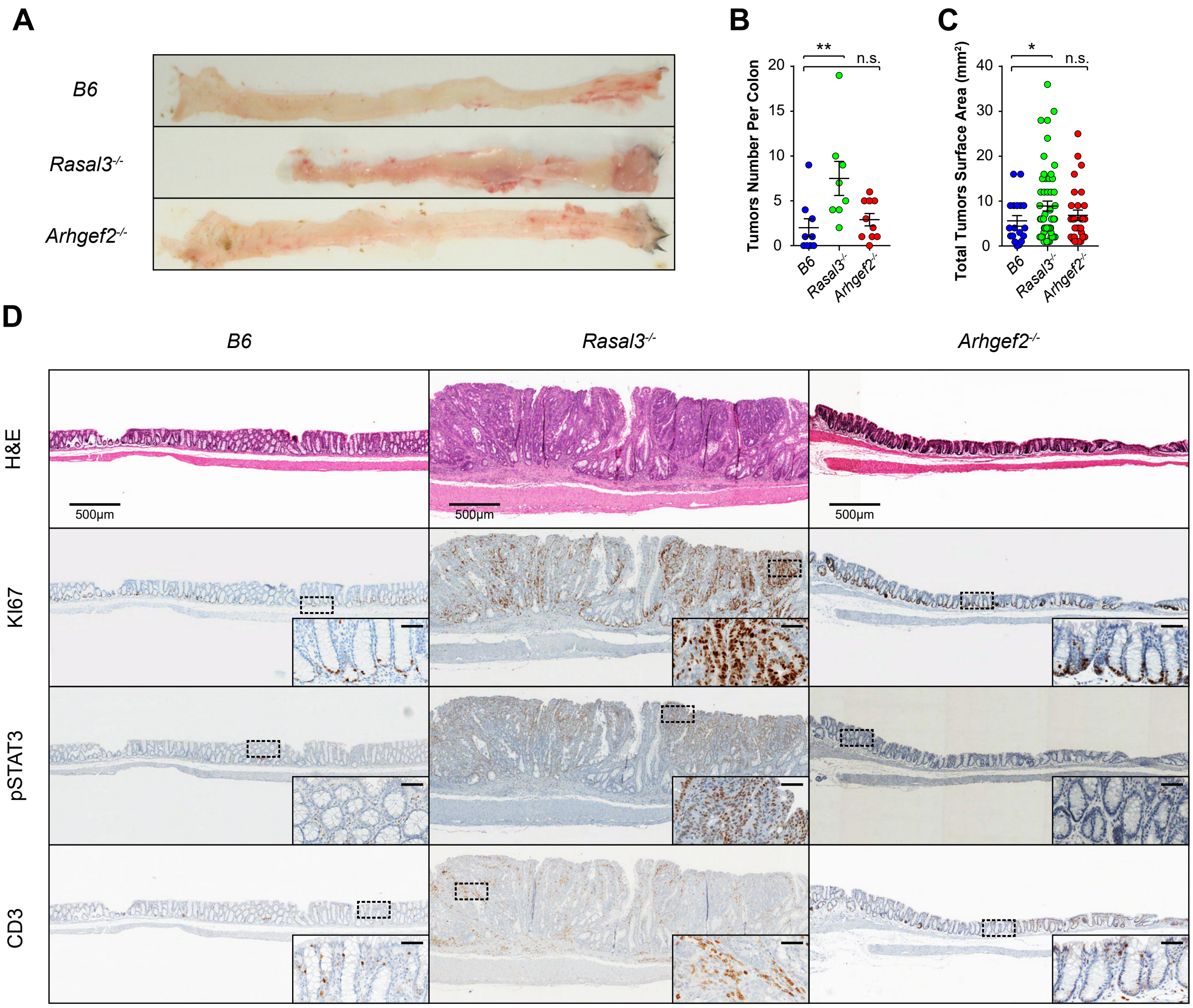

Supplemental Figure 5. Loss of RASAL3 Exacerbates Susceptibility to Colitis-Associated Colorectal Cancer (A-D) B6, *Rasal3*$^{-/-}$ and *Arhgef2*$^{-/-}$ mice were treated with azoxymethane (AOM) 7 days prior to 3 cycles of 2% DSS for 4 days at the indicated weeks. **(A)** Representative images of colons from B6, *Rasal3*$^{-/-}$ and *Arhgef2*$^{-/-}$ mice after 14 weeks. **(B-C)** Quantification of effect of treatment on the absolute number of tumors and the total tumors surface area, respectively (means ± SEM; n.s.: non-significant, $^{*}P < 0.05$, $^{**}P < 0.01$; two-tailed Student's t-test). Data from **A** to **C** are representative of 3 independent experiments. **(D)** Immunohistochemistry staining of colons of B6, *Rasal3*$^{-/-}$ and *Arhgef2*$^{-/-}$ mice at 14 weeks for the indicated markers. Inserts show magnification of regions of interest indicated by the dashed lines. Insert bar: 50µm. Data are representative of at least 3 colons per group.

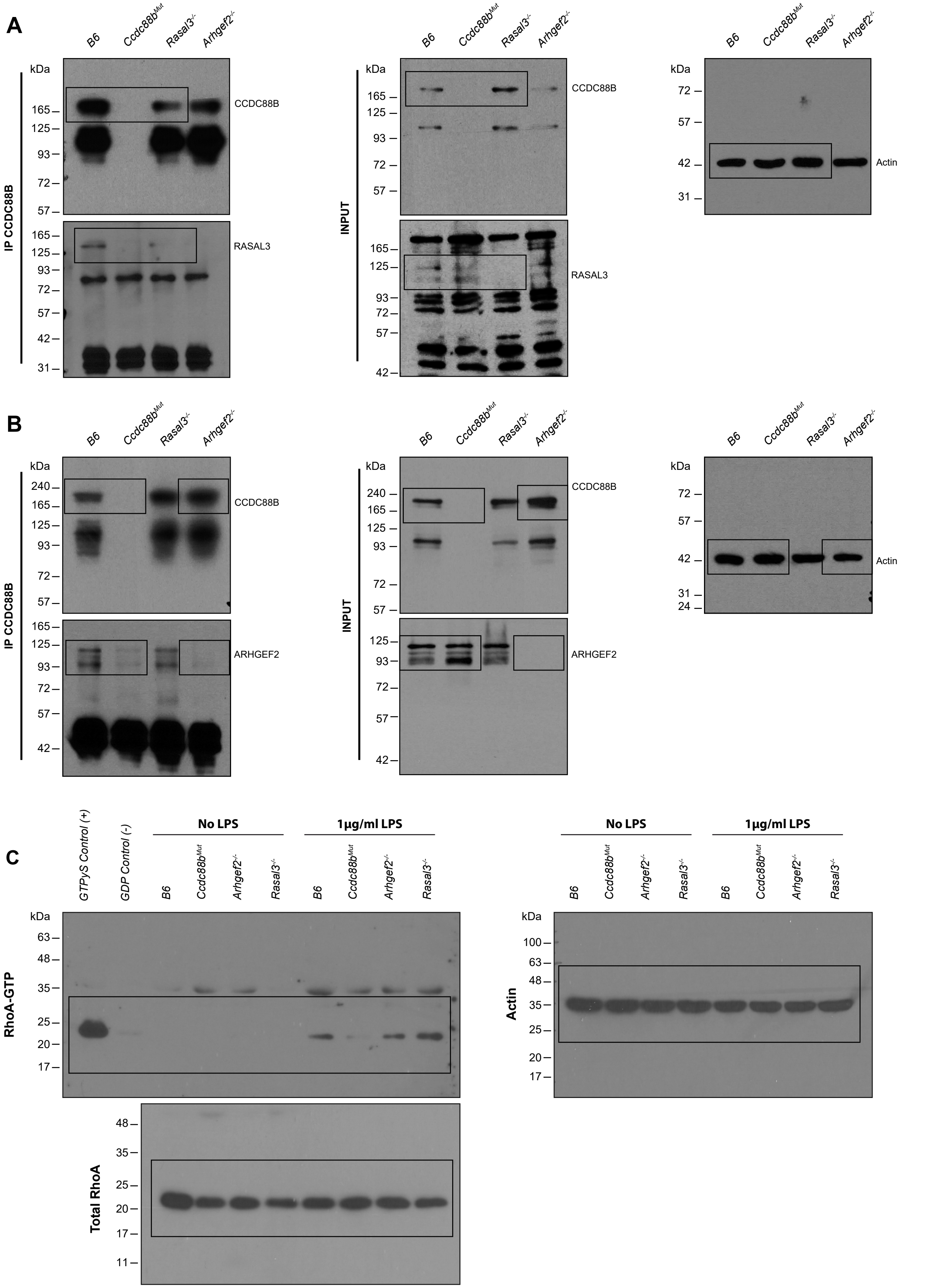

Supplemental Figure 6. Original Immunoblots for analyses. **(A-B)** From **Fig. 6E-F**, lysates from B6, *Ccdc88b*^{Mut}, *Rasal3*$^{-/-}$ and *Arhgef2*$^{-/-}$ BMDCs were immunoprecipitated with an anti-CCDC88B polyclonal antiserum, and RASAL3 or ARHGEF2 proteins were detected by immunoblotting, respectively **(C)** From **Fig. 7F**, BMDCs generated from B6, *Ccdc88b*^{Mut}, *Rasal3*$^{-/-}$ and *Arhgef2*$^{-/-}$ mutants were stimulated with LPS and protein lysate analyzed for the level of active RhoA-GTP using Rhotekin-RBD (Rho Binding Domain) beads. Identified rectangles represent the cropped image in the corresponding figure; each Western Blot is representative of 2 independent experiments.
