# Supplementary material for: CCDC88B interacts with RASAL3 and ARHGEF2 and regulates dendritic cell function in neuroinflammation and colitis
Source: Commun Biol. 2024 Jan 10;7:77. doi: 10.1038/s42003-023-05751-9 (PMC10781698; doi:10.1038/s42003-023-05751-9)
Supplement: Supplementary file 2 — Description of Supplementary Materials [file 42003_2023_5751_MOESM2_ESM.docx]

**Description of Additional Supplementary Files**

**File name:** Supplementary Data 1

**Description:** CCDC88B LC-MS/MS dataset
