# Supplementary material for: CCDC88B interacts with RASAL3 and ARHGEF2 and regulates dendritic cell function in neuroinflammation and colitis
Source: Commun Biol. 2024 Jan 10;7:77. doi: 10.1038/s42003-023-05751-9 (PMC10781698; doi:10.1038/s42003-023-05751-9)
Supplement: Supplementary file 4 — Reporting Summary [file 42003_2023_5751_MOESM4_ESM.pdf]

nature portfolio

Corresponding author(s): Philippe Gros

Last updated by author(s): Nov 15, 2023

Reporting Summary

Nature Portfolio wishes to improve the reproducibility of the work that we publish. This form provides structure for consistency and transparency in reporting. For further information on Nature Portfolio policies, see our Editorial Policies and the Editorial Policy Checklist.

Statistics

For all statistical analyses, confirm that the following items are present in the figure legend, table legend, main text, or Methods section.

n/a	Confirmed	
☐	☒	The exact sample size (*n*) for each experimental group/condition, given as a discrete number and unit of measurement
☐	☒	A statement on whether measurements were taken from distinct samples or whether the same sample was measured repeatedly
☐	☒	The statistical test(s) used AND whether they are one- or two-sided<br>*Only common tests should be described solely by name; describe more complex techniques in the Methods section.*
☐	☒	A description of all covariates tested
☐	☒	A description of any assumptions or corrections, such as tests of normality and adjustment for multiple comparisons
☐	☒	A full description of the statistical parameters including central tendency (e.g. means) or other basic estimates (e.g. regression coefficient) AND variation (e.g. standard deviation) or associated estimates of uncertainty (e.g. confidence intervals)
☐	☒	For null hypothesis testing, the test statistic (e.g. *F*, *t*, *r*) with confidence intervals, effect sizes, degrees of freedom and *P* value noted<br>*Give P values as exact values whenever suitable.*
☒	☐	For Bayesian analysis, information on the choice of priors and Markov chain Monte Carlo settings
☒	☐	For hierarchical and complex designs, identification of the appropriate level for tests and full reporting of outcomes
☐	☒	Estimates of effect sizes (e.g. Cohen's *d*, Pearson's *r*), indicating how they were calculated

Our web collection on statistics for biologists contains articles on many of the points above.

Software and code

Policy information about availability of computer code

Data collection	Immunofluorescence and brightfield microscopy data were acquire using Zen Black Imaging Software (Zeiss, version 11). Mass spectrometry data were acquire using Orbitrap Fusion Tribrid mass spectrometer (Thermo Fisher Scientific), acquiring data-dependent CID fragmentation spectra. Flow cytometry data were acquire using FACSDiva software (BD Biosciences, version 8.0.2). Single cell RNA sequencing data were collected as following: freshly sorted cells were loaded on the 10x Chromium chip; single cell capture and cDNA preparation was done according to the 10x Single Cell 3' (version 3.1) protocol, with 8000 cells targeted for capture per sample; Libraries were sequenced on the NovaSeq 6000 Sequencing System (Illumina).
Data analysis	MS data analysis was submitted to MaxQuant (Max-Planck-Institute of Biochemistry, version 1) for protein identification and label free quantification. Immunofluorescence and brightfield microscopy data were processed using ImageJ software (National Institute of health, Version 1.52p, using FIJI extension and TrackMate plugin). Data from TrackMate were further processed using a homemade MatLab script (MathWorks, Version R2017b) which be obtain from the corresponding author upon reasonable request. Flow cytometry data analyses were performed using FlowJo software (Tree Star Inc, Version 10.6.2, using tSNE plugin). Statistical analyses were performed using Prism software (GraphPad, version 5). For single cell RNA sequencing, the raw gene expression matrices were generated for each sample by the Cell Ranger, and the output was analyzed using the Seurat package.

For manuscripts utilizing custom algorithms or software that are central to the research but not yet described in published literature, software must be made available to editors and reviewers. We strongly encourage code deposition in a community repository (e.g. GitHub). See the Nature Portfolio guidelines for submitting code & software for further information.

Data

Policy information about availability of data

All manuscripts must include a data availability statement. This statement should provide the following information, where applicable:

- Accession codes, unique identifiers, or web links for publicly available datasets
- A description of any restrictions on data availability
- For clinical datasets or third party data, please ensure that the statement adheres to our policy

The MS proteomics data have been deposited to the ProteomeXchange Consortium via the PRIDE partner repository with the dataset PXD023779. The protein interactions from this publication have been submitted to the IMEx (http://www.imexconsortium.org) consortium through IntAct and assigned the identifier IM-28785. Any other data that support the findings of this study, including additional MS/MS data, are available within the article, its supplementary information and from the corresponding author upon reasonable request.

Research involving human participants, their data, or biological material

Policy information about studies with human participants or human data. See also policy information about sex, gender (identity/presentation), and sexual orientation and race, ethnicity and racism.

Reporting on sex and gender	Not applicable
Reporting on race, ethnicity, or other socially relevant groupings	Not applicable
Population characteristics	Not applicable
Recruitment	Not applicable
Ethics oversight	Not applicable

Note that full information on the approval of the study protocol must also be provided in the manuscript.

Field-specific reporting

Please select the one below that is the best fit for your research. If you are not sure, read the appropriate sections before making your selection.

☒ Life sciences ☐ Behavioural & social sciences ☐ Ecological, evolutionary & environmental sciences

For a reference copy of the document with all sections, see nature.com/documents/nr-reporting-summary-flat.pdf

Life sciences study design

All studies must disclose on these points even when the disclosure is negative.

Sample size	No sample-size calculation was performed; typical sample-size from the literature were used for each experiment, and the relevant statistical analyses can be found in the corresponding figure legend.
Data exclusions	For the LC-MS/MS analysis, abundant proteins often found in co-immunoprecipitates (CRAPome v1.1) were excluded as part of the analysis pipeline. For single cell RNA sequencing, low quality reads were filtered based on three criteria: number of detected genes per cell, number of UMIs expressed per cell and mitochondrial content, using the following threshold parameters: nGene (between 200 and 7500), nUMI (between 500 and 75,000), and percentage of mitochondrial genes expressed (< 7.5%); Doublets were then identified by finding cells expressing markers of two cell lineages simultaneously, as well as using the DoubletFinder package. No data were excluded from any other analyses.
Replication	All attempts at replication of the data were successful. The specific number of independent replicates for each experiment can be found in the corresponding figure legend.
Randomization	For all experiment, mice within the same strain were assigned randomly to either control or experimental group.
Blinding	For the mouse model of intestinal colitis, a trained pathologist scored each colon blindly as described in the method section. For immunofluorescence colon slide quantification, to remove any human bias, the 2 most distal field of view (at 10x) were systematically processed to quantify infiltration of immune cell populations using Fiji fonction Analyze Particle (Version 1.53f51). For other experiment involving mice, phenotype with clear experimental parameter, such as survival, were used to limit bias.

Reporting for specific materials, systems and methods

We require information from authors about some types of materials, experimental systems and methods used in many studies. Here, indicate whether each material, system or method listed is relevant to your study. If you are not sure if a list item applies to your research, read the appropriate section before selecting a response.

Materials & experimental systems

n/a	Involved in the study
☐	☒ Antibodies
☐	☒ Eukaryotic cell lines
☒	☐ Palaeontology and archaeology
☐	☒ Animals and other organisms
☒	☐ Clinical data
☒	☐ Dual use research of concern
☒	☐ Plants

Methods

n/a	Involved in the study
☒	☐ ChIP-seq
☐	☒ Flow cytometry
☒	☐ MRI-based neuroimaging

Antibodies

Antibodies used

Purified anti-ccdc88b and anti-Rasal3 rabbit polyclonal hyperimmune serum were produced in-house and were previously published by our groups (Kennedy et al, J Exp Med, 2014. 211(13): p. 2519-35; Muro et al, PLoS One, 2015. 10(3): p. e0119898). Other antibodies were commercially available: 1:1000 anti-FLAG (clone M2, Sigma), 1:000 anti-HA (clone F-7, Santa Cruz Biotechnology), 1:300 anti-ARHGEF2 (GEF-H1, Cell Signaling Technology),1:100 anti-Ki-67 (D3B5; Cell signaling), 1:250 anti-pSTAT3(Tyr705/D3A7, cell signaling), 1:100 anti-CD3 (SP7, Abcam), anti-CD68 (KP1, abcam), 1:200 FITC anti-NK1.1 (clone PK136, eBioscience), 1:300 PerCP-Cy5.5 anti-CD4 (clone RM4-5, Biolegend), 1:200 PE anti-TCRγδ (clone GL3, eBioscience), 1:200 PE-Dazzle 594 anti-CD3 (clone 17A2, Biolegend), 1:200 PE-Cy7 anti-CD44 (clone IM7, eBioscience), 1:300 APC anti-CD62L (clone MEL-14, eBioscience), 1:200 AlexaFluor 700 anti-CD8α (clone 53-6.7, eBioscience), 1:400 APC-Fire 750 anti-CD45 (clone 30-F11, Biolegend), 1:300 eFluor 450 anti-CD19 (clone eBio1D3, eBioscience), 1:300 FITC anti-CD64 (clone X54-5/7.1, Biolegend), 1:300 PerCP-Cy5.5 anti-Ly6G (clone 1A8, Biolegend), 1:200 PE anti-SiglecF (clone E50-2440, BDBioscience), 1:200 PE-Dazzle 594 anti-CD103 (clone 2E7, Biolegend), 1:400 PE-Cy7 anti-CD45 (30-F11, Biolegend), 1:200 APC anti-CD317 (clone 927, Biolegend), 1:800 APC-Cy7 I-A/I-E anti-MHCII (clone M5/114.15.2, Biolegend), 1:1000 BV421 anti-Ly6C (clone HK1.4, Biolegend), 1:300 BV605 anti-CD11b (clone M1/70, Biolegend), 1:300 BV785 anti-CD11c (clone N418, Biolegend), 1:400 APC anti-CD45 (clone 30-F11, eBioscience), 1:300 APC-Fire anti-CD8a (clone 53-6.7, eBioscience), 1:300 FITC anti-CD4 (GK1.5, eBioscience), 1:250 PE anti-CD25 (PC61.5,eBioscience), anti-CD45 APC (1:400, clone 30F11, Biolegend) and 1:1500 APC anti-CD45RB (C363.16A, eBioscience). Specific antibodies pairing can be found in the Material and Method section.

Validation

Purified anti-ccdc88b and anti-Rasal3 rabbit polyclonal hyperimmune serum were validated in previous paper by our groups (Kennedy et al, J Exp Med, 2014. 211(13): p. 2519-35; Muro et al, PLoS One, 2015. 10(3): p. e0119898). Anti-Arhgef2 antibodies were not able to detect any signal in Arhgef2-/- mice spleen, nor in untransfected cells (see Supp. Figure2) and were validated by the manufacturers. Other routinely used antibodies (anti-HA, anti-Flag, flow cytometry antibodies and immunohistology antibodies) were validated by the manufacturers, when applicable, and can be found on the manufacturer's website.

Eukaryotic cell lines

Policy information about cell lines and Sex and Gender in Research

Cell line source(s)

HEK293T cells from ATCC, CRL-11268

Authentication

The cell line was not authenticated

Mycoplasma contamination

The cell line was not tested for mycoplasma contamination

Commonly misidentified lines (See ICLAC register)

None

Animals and other research organisms

Policy information about studies involving animals; ARRIVE guidelines recommended for reporting animal research, and Sex and Gender in Research

Laboratory animals

Wild type C57BL/6J (B6) mice, 8 to 12 weeks of age, were obtained from the Jackson Laboratory (Bar Harbor, ME). The Ccdc88bm1PGrs mutant mouse strain (referred to as Ccdc88bMut) was generated by genome-wide chemical mutagenesis (Kennedy et al, J Exp Med, 2014. 211(13): p. 2519-35). Rasal3-/- mice were provided by Dr. H. Suzuki, from the National Center for Global Health and Medicine, Chiba (Japan), and is described elsewhere (Muro et al, PLoS One, 2015. 10(3): p. e0119898). Arhgef2-/- mice were generated by CRISPR/CAS9 by the Transgenic Core of the Life Sciences Complex at McGill University, using the experimental protocol and characterization strategy summarized in Supp. Fig.2.

Wild animals

The study did not involved wild animals

Reporting on sex

Both males and females animals were routinely used for most experiments; no difference were seen between sex in those

Reporting on sex	experiments. In some case (DSS experiments), only one sex was used; this is reported in the Material and Method section when applicable.
Field-collected samples	The study did not involved samples collected from the field
Ethics oversight	All mice were housed under specific pathogen-free conditions at the animal care facility of the Goodman Cancer Research Centre, McGill University, and the animal studies were conducted using protocols approved by the McGill Institutional Review Board (protocol number 5287) and following guidelines and regulations of the Canadian Council of Animal Care.

Note that full information on the approval of the study protocol must also be provided in the manuscript.

Flow Cytometry

Plots

Confirm that:

☒ The axis labels state the marker and fluorochrome used (e.g. CD4-FITC).

☒ The axis scales are clearly visible. Include numbers along axes only for bottom left plot of group (a 'group' is an analysis of identical markers).

☒ All plots are contour plots with outliers or pseudocolor plots.

☒ A numerical value for number of cells or percentage (with statistics) is provided.

Methodology

Sample preparation	In some experiments, colons from mice were dissociated with Lamina Propria Dissociation Kit (Miltenyi Biotec), according to the manufacturer instructions, resulting in a single-cell suspension. In other experiments, mice spleens were harvested, processed into single cell suspensions by crushing though a cell 70µm cell strainer and red blood cells lysed using ammonium chloride containing buffer (ACK; Fisher scientific). In all case, cell suspensions were stained with vital dye Zombie Aqua dye (1:400 dilution, Biolegend), then surface stained with fluorescently labeled antibodies, and finally fixed using Cytofix Fixation Buffer (BD Biosiences) accordingly to the manufacturer protocol. Additional information can be found in the Materials and Methods section.
Instrument	4 lasers (405/488/561/633 nm) LSR Fortessa flow cytometer (BD Biosciences) and 3 lasers (405/488/640 nm) FACSAriaII cell sorter (BD Biosciences).
Software	Flow cytometry data were acquire using FACSDiva software (BD Biosciences, version 8.0.2), and analysed using FlowJo software (Tree Star Inc, Version 10.6.2, using tSNE plugin).
Cell population abundance	Not applicable
Gating strategy	For all experiments, debris were excluded based on low FSC-A/SSC-A signal and doublet excluded based on FSH-H/FSH-A. Primary gating was also performed on CD45+ viable cell, before proceeding to tSNE visualization or further gating as described in the Materials and Methods section. A full gating strategy can be found in Supp. Fig. 3.

☒ Tick this box to confirm that a figure exemplifying the gating strategy is provided in the Supplementary Information.
